# The mammosphere-derived epithelial cell secretome modulates neutrophil functions in the bovine model

**DOI:** 10.3389/fimmu.2024.1367432

**Published:** 2024-06-27

**Authors:** Rebecca M. Harman, Anja Sipka, Kelly A. Oxford, Leane Oliveira, Lucas Huntimer, Daryl V. Nydam, Gerlinde R. Van de Walle

**Affiliations:** ^1^ Baker Institute for Animal Health, College of Veterinary Medicine, Cornell University, Ithaca, NY, United States; ^2^ Department of Population Medicine and Diagnostic Sciences, College of Veterinary Medicine, Cornell University, Ithaca, NY, United States; ^3^ Elanco Animal Health, Indianapolis, IN, United States; ^4^ Department of Public and Ecosystem Health, Cornell University, Ithaca, NY, United States

**Keywords:** bovine, mammosphere-derived epithelial cells, neutrophils, secretome, host-directed immunotherapy

## Abstract

**Background:**

Innovative therapies against bacterial infections are needed. One approach is to focus on host-directed immunotherapy (HDT), with treatments that exploit natural processes of the host immune system. The goals of this type of therapy are to stimulate protective immunity while minimizing inflammation-induced tissue damage. We use non-traditional large animal models to explore the potential of the mammosphere-derived epithelial cell (MDEC) secretome, consisting of all bioactive factors released by the cells, to modulate host immune functions. MDEC cultures are enriched for mammary stem and progenitor cells and can be generated from virtually any mammal. We previously demonstrated that the bovine MDEC secretome, collected and delivered as conditioned medium (CM), inhibits the growth of bacteria *in vitro* and stimulates functions related to tissue repair in cultured endothelial and epithelial cells.

**Methods:**

The immunomodulatory effects of the bovine MDEC secretome on bovine neutrophils, an innate immune cell type critical for resolving bacterial infections, were determined *in vitro* using functional assays. The effects of MDEC CM on neutrophil molecular pathways were explored by evaluating the production of specific cytokines by neutrophils and examining global gene expression patterns in MDEC CM-treated neutrophils. Enzyme linked immunosorbent assays were used to determine the concentrations of select proteins in MDEC CM and siRNAs were used to reduce the expression of specific MDEC-secreted proteins, allowing for the identification of bioactive factors modulating neutrophil functions.

**Results:**

Neutrophils exposed to MDEC secretome exhibited increased chemotaxis and phagocytosis and decreased intracellular reactive oxygen species and extracellular trap formation, when compared to neutrophils exposed to control medium. C-X-C motif chemokine 6, superoxide dismutase, peroxiredoxin-2, and catalase, each present in the bovine MDEC secretome, were found to modulate neutrophil functions.

**Conclusion:**

The MDEC secretome administered to treat bacterial infections may increase neutrophil recruitment to the site of infection, stimulate pathogen phagocytosis by neutrophils, and reduce neutrophil-produced ROS accumulation. As a result, pathogen clearance might be improved and local inflammation and tissue damage reduced.

## Introduction

Worldwide, more than 700,000 people die each year from drug-resistant strains of common bacteria (1). In the US alone, greater than 2 million infections per year are caused by bacteria that are resistant to first line antibiotics, costing the health care system in excess of $20 billion (2, 3). This increasing burden of drug-resistant bacteria on physical and economic health, combined with the fact that many host-pathogen interactions are not satisfactorily resolved by antimicrobial therapy alone, prompt the need for new strategies to treat bacterial infections (4, 5).

One approach is to pursue host-directed immunotherapies (HDTs), aimed to make natural bacterial defense more effective and efficient (4, 6, 7). When developing these therapies, one must consider the multifaceted and complex interactions of immune cells with pathogens and with each other. While immune responses may serve to protect against diseases initiated by pathogens, they can cause inflammation induced tissue damage in the process (8). An overarching goal of HDT development is to augment host antimicrobial defense mechanisms while attenuating damaging cascades that lead to tissue injury (4, 8). Various methods of targeting the host immune system, spanning disciplines, may achieve these goals. Nanotherapeutics, the application of drugs and devices in the size of 1–100 nm, can facilitate direct and site-specific delivery of drugs or blocking agents to infected areas (7, 9). Nucleic acids and antibodies can be used to stimulate or repress specific gene and protein targets, respectively, to fine tune responses of immune cells to pathogens (8), and small molecules, low molecular weight compounds of less than 1 kD, can be delivered for immunomodulatory cell and gene therapy (10).

Tissue specific adult stem cells (TSASCs) from veterinary species, including cattle, have been studied as potential biologic therapies for immune induced pathologic conditions such as inflammation (11–13). The predominant immunomodulatory functions of TSASCs are thought to occur via secreted bioactive factors that interact with target cells in the recipient/patient (11, 14, 15). Secreted bioactive factors from TSASCs include small molecules, nucleic acids, peptides and proteins, that are either soluble or associated with lipid bound extracellular vesicles (14, 16–18), and are collectively called the secretome. While efforts have been made to identify individual bioactive factors in the secretome of TSASC that interact with target cells to elicit specific responses, evidence suggests that treatment with the complete secretome yields more desirable effects than treatment with individual components (16, 19).

One type of TSASCs are mammosphere-derived epithelial cells (MDECs) which can be isolated from a wide range of domestic and wild mammals (20, 21). MDEC cultures are enriched for mammary stem and progenitor cells, and conditioned medium (CM) collected from MDEC cultures serves as a source of the MDEC secretome that can be used to deliver bioactive factors to target cells *in vitro* (22, 23). We have previously shown that the CM from bovine MDECs promotes angiogenesis and epithelial cell migration, and contains factors associated with defense and immunity (23).

The current study was designed to expand on these data by performing *in vitro* experiments to determine if bovine MDEC CM modulates neutrophil functions. Neutrophils are short lived, mobile, phagocytic cells that are part of the innate immune system. They are the first line of defense against bacterial infection and can contribute to inflammation induced tissue damage (24). Bacterial invasion of tissues and subsequent inflammation can dramatically increase influx of neutrophils, often within a few hours post infection. Neutrophils directly attack microorganisms by (i) phagocytosis, the ingestion and killing of bacteria, (ii) degranulation, which releases soluble anti-microbial compounds into the surrounding tissues, and (iii) generation of neutrophil extracellular traps (NETs), web-like structures comprised of chromatin and serine proteases that physically trap bacteria, exposing them to high local concentrations of anti-microbial compounds (25). While these processes are effective at pathogen elimination during infection, they often occur at the expense of surrounding tissues, which can be damaged by robust neutrophilic responses (26, 27).

An HDT such as MDEC CM, that could regulate neutrophil interactions with pathogens and host tissues during bacterial infection, could reduce the negative effects of common and economically impactful pathogen-induced diseases of dairy cattle. Two such diseases are mastitis and metritis. Mastitis is an inflammatory response of tissue in the mammary gland caused by bacteria and their byproducts, as well as neutrophil activity. When pathogens invade the mammary gland, epithelial cells and macrophages release chemokines that attract neutrophils from the blood into mammary tissues. Neutrophils in the tissue will engulf and destroy pathogens by releasing reactive oxygen species (ROS) and granular enzymes, processes which also damage epithelial cells and hinder mammary function (26, 28, 29). Metritis, a postpartum disease of dairy cattle, is caused by a mixture of microorganisms and characterized by inflammation and a fetid discharge from the uterus. Neutrophils are the main line of defense against bacteria in the uterus, and likewise, neutrophil secreted products can injure tissues and trigger a dysregulated immune response as a byproduct of antimicrobial activity (30–35).

The aims of this study were to determine if the bovine MDEC secretome modulates neutrophil functions, and if so, what bioactive components of the secretome are responsible for the observed effects. A long-term objective of this work is to determine if the MDEC secretome from various mammals can be used as an HDT against bacterial infections.

## Materials and methods

Catalog numbers for non-antibody reagents can be found in **Supplementary Table 1**. Information on antibodies, including catalog numbers where applicable, are presented in **Table 1**. All reagent concentrations written in this section represent the final concentrations used in cell cultures.

**Table 1:** Antibodies used for immunocytochemistry assays, fluorescent bead-based multiplex assays and Western blots.

Target	Host	Clone	Dilution	Conjugate	Manufacturer	Catalog #
Myeloperoxidase	Mouse	266–6K1	100 µg/ml	none	Santa Cruz Biotechnology	SC-52707
Neutrophil Elastase	Mouse	F-1	200 µg/ml	none	Santa Cruz Biotechnology	SC-55548
Citrullinated Histone H3	Mouse	RM1001	500 µg/ml	none	Abcam	Ab281584
**Inteferon γ**	Mouse	CC302	1:200	biotin	BioRad	MCA1783B
**Interleukin 10**	Mouse	492–2	1:500	biotin	in-house	NA^a^
**Tumor necrosis factor α**	Mouse	65–2	1:200	biotin	in-house	NA
Mouse IgG Isotype Control	Mouse	none	200 µg/ml	none	Abcam	Ab18443
Rabbit IgG Isotype Control	Rabbit	none	500 µg/ml	none	Abcam	Ab172730
Mouse IgG (H+L)	Goat	polyclonal	1:100	Alexa 488	Jackson ImmunoResearch	111–545-146
C-X-C motif chemokine ligand 6	Rabbit	EPR22310–196	1:1000	none	Abcam	ab243097
Insulin like growth factor 2	Rabbit	polyclonal	1:1000	none	Abcam	ab226989
Superoxide Dismutase	Rabbit	polyclonal	1:200	none	Abcam	ab83108
Peroxiredoxin 2	Rabbit	polyclonal	1:200	none	Abcam	ab2269922
Catalase	Rabbit	polyclonal	1:200	none	Abcam	ab16731
Fibronectin	Rabbit	polyclonal	1:1000	none	Sigma Aldrich	F3648
β-Actin	Rabbit	polyclonal	1:1000	none	Abcam	Ab8227
Rabbit IgG (H+L)	Goat	polyclonal	1:20,000	HRP^b^	Jackson ImmunoResearch	111–035-144

^a^NA, not applicable; ^b^HRP, horseradish peroxidase.

### Cell isolation, culture, and conditioned medium collection

Bovine mammosphere-derived epithelial cells (MDECs) were isolated from the udder tissue of Holstein cows after euthanasia for reasons unrelated to this study, and cultured according to our protocols for generating mammary epithelial cultures enriched for stem and progenitor cells (20). CM containing all factors secreted by MDECs was collected as described previously for viability, chemotaxis, phagocytosis and reactive oxygen species (ROS) assays, as well as for treating neutrophils used for RNA deep sequencing (RNAseq) (23), using RPMI medium (Corning Incorporated, Corning, NY) + 2% filtered fetal bovine serum (FBS) (R&D Systems, Minneapolis, MN) as the base medium. CM for assays designed to evaluate features associated with neutrophil extracellular traps (NETs), including neutrophil elastase (NE) activity assays, immunofluorescent (IF) antibody binding assays, and extracellular versus intracellular DNA visualization, was generated using RPMI + 1% bovine serum albumin (BSA) (Sigma Aldrich, St. Louis, MO), as FBS has been documented to affect NET stability (36) and serum free culture has been shown to induce NET formation (37).

Blood collection for neutrophil isolation and serum collection was approved by the Cornell Institutional Animal Care and Use Committee (IACUC # 2014–0038). We obtained 40 ml peripheral blood by puncture of the coccygeal vessels of lactating Holstein cows (n = 27) into evacuated tubes containing ethylenediaminetetraacetic acid (EDTA) and another 10 ml blood from the same animal into serum tubes. For serum collection, tubes were incubated at room temperature (RT) for 30 min, then centrifuged at 2000 x g for 10 min at 4°C. Serum was removed from clot, and frozen in aliquots at -20°C. To separate neutrophils, blood was removed from tubes containing EDTA, diluted 1:1 with phosphate-buffered saline (PBS), layered over Ficollpaque Plus (GE Healthcare, Chicago, IL), and centrifuged at 1000 x g for 30 min at RT with no brake. Plasma, interphases, and Ficollpaque Plus, were removed and discarded. Remaining cell pellets consisting primarily of red blood cells (RBCs) and neutrophils were manually dislodged and RBCs were lysed with a 0.2% NaCl solution. An equal volume of a 1.6% NaCl solution was added, and cells were mixed gently by inversion. Cells were washed by centrifugation at 250 x g for 10 min at 4°C. Pellets were dislodged and RBC lysis was repeated. Neutrophils were resuspended in RPMI medium with 1% HEPES (Corning Incorporated), mixed 1:1 with trypan blue (Corning Incorporated) to visually assess viability, and counted.

### Viability and apoptosis assays

To determine if MDEC CM is toxic to neutrophils in the planned functional experiments, neutrophils were incubated with RPMI + 2% FBS (control), MDEC CM, or 100 µM busulfan (positive control) at 37°C with 5% CO_2_ for 1 hour (h). Cells were then washed with RPMI + 2% FBS by centrifugation at 300 x g for 5 min at RT and maintained for 5 h in RPMI + 2% FBS. To distinguish live from dead cells, 0.5 µg/ml propidium iodide (PI) was added just prior to analysis, at approximately 6 h post isolation. Caspase activity assays were performed using TF2-VAD-FMK as a fluorescent indicator for activated caspase-1, -3, -4, -5, -6, -7, -8 and -9 in apoptotic cells, according to manufacturer’s instructions (Abcam, Waltham, MA). PI was added to cells immediately prior to analysis, again at about 6 h post isolation. Fluorescence for both assays was analyzed on a BD LSRFortessa X-20 flow cytometer with FACSDiva software (BD Biosciences, San Jose, CA). FlowJo software (FlowJo LLC, Ashland, OR) was used to calculate the mean fluorescent intensity (MFI) of labeled neutrophils. The gating scheme for these, and other flow cytometry assays is shown in **Supplementary Figure 1A**, and MFI data used to calculate “MFI (% Control)” for this and all other flow cytometry-based assays are presented in **Supplementary Table 2**.

### Chemotaxis assays

For chemotaxis assays, 24-well tissue culture plate wells were fitted with 12 mm, circular glass coverslips and 6.5 mm transwell inserts with a 3.0 µm pore size (Corning Incorporated). RPMI + 2% FBS (control), MDEC CM, 50 ng/ml bovine interleukin-8 (IL8) (Kingfisher Biotech, Saint Paul, MN) or 10 ng/ml bovine C-X-C motif chemokine ligand 6 (CXCL6) (Novus International, St. Louis, MO) diluted in RPMI + 2% FBS were added to the bottoms of the wells, and 50,000 neutrophils were added to the tops of the inserts. Plates were incubated and 37°C with 5% CO_2_ for 1 h, inserts were removed, and plates were centrifuged at 250 x g for 5 min at RT to collect neutrophils that had migrated on the surfaces of the coverslips. Treatment media were gently removed, and neutrophils that had migrated were fixed to coverslips with cold 70% EtOH and stained with hematoxylin and eosin (Sigma Aldrich). Coverslips were removed from wells and mounted on glass slides for imaging. Brightfield images of ten random fields per coverslip were captured using an Olympus CKX41 inverted microscope and Infinity 2 digital camera (Olympus Corporation, Center Valley, PA), and neutrophils were manually counted, in a blinded manner, from images.

### Phagocytosis assays

pHrodo green *Escherichia (E.) coli* bioparticles (Thermo Fisher Scientific, Waltham, MA) were diluted with 2 ml RPMI + 1% HEPES to a concentration of 1 mg/ml, according to manufacturer’s instructions. Bioparticles were opsonized with 1% cow serum (20 µl) that had been collected on site, by incubation at 37°C for 1 h, while gently rocking. Neutrophils were distributed into 4 ml tubes, with 1 x 10^6^ cells per tube. Treatments consisting of either RPMI + 2% FBS (control), MDEC CM, 20 ng/ml bovine granulocyte-macrophage colony-stimulating factor (GM CSF) or 50 ng/ml bovine insulin-like growth factor 2 (IGF2) (both from Kingfisher Biotech, and diluted in RPMI + 2% FBS), were added, and cells were incubated for 1 h at 37°C. Cells were washed with RPMI + 2% FBS by centrifugation, 100 µl bioparticles were added, and cells were incubated for 1 h at 37°C. Cells were washed with RPMI + 2% FBS by centrifugation, and resuspended in PBS. A small aliquot of cells with particles (20 µl) was removed and held for microscopic visualization, and 0.5 µg/ml PI was added to the remainder. Neutrophils, neutrophils with PI or bioparticles, neutrophils with PI and bioparticles, and bioparticles alone, were analyzed on the BD LSRFortessa X-20 flow cytometer with FACSDiva software. FlowJo software was used to calculate MFI according to the gating scheme depicted in **Supplementary Figure 1A**. The pHrodo-labeled bioparticles are pH-sensitive, showing little fluorescent signal at the neutral pH of culture medium or physiologic buffers but bright fluorescence in the acidic environment of the neutrophil phagosome. This feature reduces the risk of detecting free particles or particles adhered to the outside of cells in the fluorescent channel. Cells removed for visualization were counterstained with the nuclear indicator 4’6-diamidino-2-phenylindole (DAPI, Sigma Aldrich) at 10 µg/ml, transferred to slides, and imaged using a 100x objective (UPlanApo, 100x/1.50 oil HR, ∞/0.13–0.19/OFN22) on an Olympus FV3000 confocal microscope with cellSens imaging software (Olympus Corporation).

### Reactive oxygen species assays

Neutrophils were distributed into 4 ml tubes, with 1 x 10^6^ cells per tube. Treatments consisting of either RPMI + 2% FBS (control), MDEC CM, 500 U/ml superoxide dismutase (SOD), 20 µg/ml peroxiredoxin 2 (PII), or 250 U/ml catalase (CAT) (all from Sigma Aldrich, and diluted in RPMI + 2% FBS) were added and cells were incubated for 1 h at 37°C. Cells were washed with RPMI + 2% FBS by centrifugation and 7.5 µM 2’,7’-dichlorodihydrofluorescein diacetate (H2DCFDA) (Sigma Aldrich), diluted in RPMI + 1% HEPES, was added. After a 15 min incubation at 37°C, cells were washed again before adding the neutrophil stimulant, phorbol myristate acetate (PMA) (Sigma Aldrich) at 25 ng/ml in RPMI + 1% HEPES. Cells were again incubated for 15 min at 37°C, put on ice to stop reactions. Cells were washed with RPMI + 1% HEPES by centrifugation and resuspended in PBS. A small aliquot of cells (20 µl) was removed and held for visualization, and 0.5 µg/ml PI was added to the remainder. Neutrophils, neutrophils with PI or H2DCFDA, and neutrophils with PI and H2DCFDA were analyzed on the BD LSRFortessa X-20 flow cytometer with FACSDiva software. FlowJo software was used to calculate MFI of samples and control cells according to the gating scheme shown in **Supplementary Figure 1A**. Cells removed for visualization were counterstained with 10 µg/ml DAPI, transferred to slides, and imaged using the 100x objective of the Olympus FV3000 confocal microscope with cellSens imaging software.

### Neutrophil elastase activity assays

NE activity assays were carried out, according to manufacturer’s instructions (Abcam). Briefly, 1 x 10^5^ neutrophils in 100 µl NET Assay Buffer (RPMI + 1% BSA + 1 mM CaCl_2_) were added to 24-well plate wells. 800 µl RPMI + 1% BSA or MDEC CM was added to each well and incubated for 1 h at 37°C. Treatment media were removed, cells were gently rinsed with RPMI + 1% BSA and neutrophils were treated with 25 ng/ml PMA and incubated for 4 h at 37°C. Neutrophils were rinsed twice with NET Assay Buffer before the addition of S7 nuclease, to digest NET DNA, for 45 min at 37°C. Supernatants from wells were transferred to microfuge tubes and ethylenediaminetetraacetic acid (EDTA, Sigma Aldrich) was added to a final concentration of 10 mM to inactivate the nuclease. Tubes were centrifuged to remove debris and supernatants were transferred to 96-well plate wells. NE substrate was added to each well and plates were incubated for 2 h at 37°C. Absorbance at 405 nm was measured using a Tecan Infinite 200 Pro plate reader (Tecan U.S., Morrisville, NC). NE standards of known concentrations were included on each plate. Absorbances versus concentrations of standards were plotted using GraphPad Prism (GraphPad Software, San Diego, CA), and sample concentrations were interpolated based on best fit values.

### Immunofluorescence assays

For fluorescent antibody binding assays to visualize the effects of MDEC CM on proteins associated with neutrophil extracellular trap (NET) formation, neutrophils were plated at 1 x 10^5^ cells per well 24-well plate wells fitted with glass coverslips and incubated with either RPMI + 1% BSA (control) or MDEC CM, each +/- 25 ng/ml PMA for 1 h at 37°C. Treatment media were removed, cells were gently rinsed with PBS, and then fixed and permeabilized as previously described (38). For antibody binding, cells were blocked for 15 min with PBS + 1% BSA and primary antibodies against neutrophil elastase (NE), a serine protease secreted by neutrophils during inflammation and NET production (39), myeloperoxidase (MPO), an enzyme required for NET formation (40), and citrullinated histone H3 (H3), a histone modification associated with NETs (41), were diluted in PBS as described in **Table 1**, were added. Neutrophils were incubated with primary antibodies or isotype control antibodies for 1 h at RT, rinsed 3 x 5 min with PBS, and then incubated with Alexa 488-conjugated secondary antibodies, diluted in PBS as described in **Table 1**, for 1 h at RT. Neutrophils were rinsed 3 x 5 min with PBS and the DNA stain SYTOX^™^ Orange (Thermo Fisher Scientific) was added at 1:30,000 in PBS. After a 5 min incubation at RT, cells were briefly rinsed in PBS and coverslips were transferred to slides. Cells were imaged using the 100x objective of an Olympus FluoView FV3000 confocal microscope (Olympus Corporation). The integrated density of pixels representing specific antibody binding in ten images per condition was determined using Fiji/ImageJ software (42) and normalized to account for number of cells per image.

### Extracellular versus intracellular DNA visualization

To visualize neutrophil extracellular versus intracellular DNA, reagents from a NETosis imaging assay kit (Cayman Chemical, Ann Arbor, MI) were used, according to manufacturer’s instructions. Briefly, neutrophils were plated at a concentration of 80,000 cells per well were plated in 48-well plates and treated with RPMI + 1% BSA (control) or MDEC CM, with or without 25 ng/ml PMA for 1 h. 5 µM permeable nuclear red reagent was added and cells were incubated for 15 min at 37°C in the dark. Cells were rinsed with PBS by centrifugation and resuspended in NETosis Imaging Buffer with 5 µM extracellular green reagent, before imaging at 0, 3, 6 and 9 h using the 20x objective on a ZOE fluorescent cell imager (Bio Rad, Hercules, CA). The integrated density of pixels representing extracellular and intracellular DNA in each image taken at each timepoint was determined using Fiji/ImageJ software, and data were expressed as intracellular/extracellular DNA.

### Enzyme-linked immunosorbent assays

ELISAs were used to detect and quantify the concentrations of complement components C3 (C3) and C5a (C5a), bovine CXC motif chemokine ligand 6 (CXCL6), insulin like growth factor 2 (IGF2), superoxide dismutase 1 (SOD), peroxiredoxin 2 (PII) and catalase (CAT) in CM, as per manufacturer’s instructions (MyBioSource, San Diego, CA). Absorbance was measured on the Tecan Infinite 200 Pro plate reader (Tecan) at 450 nm. Absorbances versus concentrations of standards were plotted using GraphPad Prism (GraphPad Software) and sample concentrations were interpolated based on best fit values. For analysis of MDEC CM, CM from 3 MDEC lines was tested in duplicate in each assay and assays were repeated 3 times. RPMI + 2% FBS was included as control medium.

### Fluorescent bead-based multiplex assay for cytokine measurements

The multiplex assay was performed, as previously described (43). All antibodies used are monoclonal antibodies (mAbs) of IgG1 isotypes and were generated in mice. Equine antibodies used have been shown to be cross-reactive with bovine proteins (43). If not indicated otherwise, mAbs were produced in house (43–46). Briefly, fluorescent beads were coupled with bovine interleukin-10 (IL10) mAb 130, equine interferon gamma (IFNγ) mAb 3, and tumor necrosis factor alpha (TNFα) mAb 197–1. Coupled beads were individually sonicated and a bead mixture was prepared in PBS with 1% BSA and 0.05% sodium azide (PBN) with a final concentration of 5 x 10^3^ beads each. The cytokine standard mix containing recombinant fusion proteins for all 3 targets was prepared in 5-fold dilutions in PBN. Standards, samples, or PBN (blank values) were added to 96-well filter plates (MultiScreen HTS, Millipore, Burlington, MA) followed by the bead mixture. After 30 min incubation at RT in the dark, plates were washed 3 times with a plate washer (ELx50, Bio Tek, Winooski, VT), conditions that were also used for following incubation and wash steps. Subsequently, a mixture of biotinylated mAbs in PBN was added consisting of equine IL10 mAb 492–2, bovine IFNγ mAb CC-302 (Bio Rad), and bovine TNFα mAb 65–2. After another incubation and wash step, the reporter dye streptavidin R-PE (Thermo Fisher) was added, followed by a final incubation and wash. PBN was added to the plate and plates were read in an automated reader (Luminex 200, Bio Rad). Data were reported as median fluorescence intensity, and the standard curves were fitted with a 5-parameter logistic regression by the Bio-Plex software (Bio-Plex Manager 6.2, Bio Rad).

### RNA sequencing

Neutrophils for RNA deep sequencing were incubated with RPMI + 2% FBS (control) or MDEC CM at 37°C for 1 h. Cells were then rinsed with PBS by centrifugation at 2000 x g for 10 min at RT and PBS was removed before cell pellets were snap frozen in liquid nitrogen and held at -80°C until RNA was extracted. Total RNA for sequencing was extracted with a QIAGEN RNeasy Plus Mini Kit as described previously (38), and RNA quantity was measured using a NanoDrop spectrophotometer (Thermo Fisher). The concentration of total RNA was confirmed using a Qubit RNA HS kit (Thermo Fisher), and a Fragment Analyzer (Agilent, Santa Clara, CA) was used to determine adequate RNA integrity. PolyA+ RNA was isolated with the NEBNext Poly(A) mRNA Magnetic Isolation Module (New England Biolabs, Ipswich, MA). UDI-barcoded RNAseq libraries were generated with the NEBNext Ultra II RNA Library Prep Kit (New England Biolabs). Each library was quantified with a Qubit dsDNA HS kit (Thermo Fisher) and the size distribution was determined with a Fragment Analyzer (Agilent) prior to pooling. Libraries were sequenced on an Illumina instrument. At least 20M reads were generated per library. Reads were trimmed for low quality and adaptor sequences with TrimGalore v0.6.0 (47), a wrapper for cutadapt (48, 49) and fastQC (50). [Parameters: -j 1 -e 0.1 –nextseq-trim=20 -O 1 -a AGATCGGAAGAGC –length 50 –fastqc] Reads were mapped to the reference genome/transcriptome *Bos Taurus* ARS-UCD1.2 (51) using STAR v2.7.0e (52) [Parameters:–outSAMstrandField intronMotif, –outFilterIntronMotifs RemoveNoncanonical, –outSAMtype BAM SortedByCoordinate, –quantMode GeneCounts] SARTools and DESeq2 v1.26.0 were used to generate normalized counts and statistical analysis of differential gene expression (53–55). [Parameters: fitType parametric, cooksCutoff TRUE, independentFiltering TRUE, alpha 0.05, pAdjustMethod BH, typeTrans VST, locfunc median].

### Double-stranded RNA-mediated interference

Custom Silencer Select siRNA targeting bovine CXC motif chemokine ligand 6 *(CXCL6)*, insulin like growth factor 2 *(IGF2)*, superoxide dismutase 1 *(SOD)*, peroxiredoxin 2 *(PII)* and catalase *(CAT)* were designed by Thermo Fisher. Silencer Select Negative Control #2 siRNA (Thermo Fisher) was confirmed to be non-complementary to all bovine protein coding genes using BLAST (56) and was included as a negative control. Cells were plated and transfected, as previously described (57), and base medium consisting of RPMI + 2% FBS was added 24 h post-transfection to generate CM, as described above.

### Reverse transcription-polymerase chain reaction

SYBR green-based RT-PCR was performed to determine relative expression levels of transcripts of interest and data were analyzed, as previously described (22, 58). Glyceraldehyde 3-phosphate dehydrogenase (*GAPDH)*, an accepted reference gene for bovine cells (59), was used to normalize cDNA input across samples. PCR primer sequences are listed in **Table 2**.

**Table 2:** Primers used for reverse transcriptase (RT)-PCR.

Gene Name	Gene Symbol	Forward primer (5’-3’)	Reverse primer (5’-3’)
CXC motif chemokine ligand 6	*CXCL5*	CCTCTGGACCCACTGAAGAC	CAAGGGCAAGCATAGATTCC
Insulin like growth factor 2	*IGF2*	CAGAGTGAGGAACGGTTTGG	CTCCCCAGATCTTGCAGAAG
Superoxide dismutase 1	*SOD1*	ACCAGATGACTTGGGCAGAG	ATTACACCACAGGCCAAACG
Peroxiredoxin 2	*PRDX2*	CCACGGAGATCGTAGCTTTC	TTTCCTGGGAGTGTTGATCC
Catalase	*CAT*	GAACTGTCCCTACCGTGCTC	AAGTGGGTCCTGTGTTCCAG
Glyceraldehyde-3-phoshate dehydrogenase	*GAPDH*	CACAGTCAAGGCAGAGAACG	TACTCAGCACCAGCATCACC

### Western blotting

WB was used to determine the efficacy of siRNA silencing of genes in bovine MDECs, as previously described (38, 57). Antibodies used for WB are shown in **Table 1**. Images of blots were captured on a ChemiDoc Touch Imaging System and band intensities of proteins of interest relative to reference protein bands were determined using Image Lab software (both from Bio Rad). We and other groups have previously used ubiquitously expressed secreted and structural proteins as references for equal loading across CM samples in WB (57, 60, 61). To determine a suitable reference protein for the bovine MDEC CM samples in this study, CM from 3 bovine MDEC lines were run on a WB, each as undiluted, diluted 1:2, and diluted 1:4. Membranes were probed with either an anti-fibronectin or anti-β-actin antibody. Densities of bands representing each protein were consistent across undiluted samples and dilution of samples resulted in lower band densities (**Supplementary Figures 1B, C**). We chose fibronectin as the reference protein based on the following. Blots to determine the efficacy of siRNA silencing were first probed with antibodies against proteins of interest, stripped, and then probed with the reference protein antibody. Since fibronectin bands were visible at 150 kD, well above the bands representing the proteins of interest, thus greatly reducing the likelihood of misinterpreting the data if stripping was not entirely effective.

### Statistical analysis

Initial viability, apoptosis, chemotaxis, phagocytosis, and ROS accumulation assays were run as 3 experiments, each on a different day (i.e., experiment 1, 2, and 3; **Supplementary Figure 2A**). For each experiment, CM from MDECs generated from 3 different cows (i.e., MDEC lines 1, 2 & 3) was used and tested on neutrophils isolated from the blood of 3 different cows (e.g., neutrophil a, b & c for experiment 1; **Supplementary Figure 2A**). Within an experiment, data from the 3 neutrophil isolations treated with CM from each MDEC cell line were averaged to create the data points shown on the cell line specific graphs (i.e., MDEC Cell Line 1, 2 & 3 Graph; **Supplementary Figure 2A**). Viability and apoptosis data from all 3 MDEC cell lines were compiled on single graphs. To analyze cytokines secreted by neutrophils, CM from MDECs generated from 3 different cows (i.e., MDEC lines 1, 2 & 3) was used and tested on neutrophils isolated from the blood of 3 different cows. For assays consisting of three or more groups, ANOVAs were used to determine if the means between groups were significantly different. ANOVAs were followed by Dunnett’s multiple comparisons tests set up to compare the mean of each test group to the mean of the control group. For assays consisting of two groups, Student’s t-tests were used to compare the means of the groups.

NE activity, IF assays, and assays to determine ratios of intracellular/extracellular DNA, in addition to the RT-PCR, WB, chemotaxis, phagocytosis, and ROS production assays designed to determine which bioactive factors in MDEC CM were responsible for functional effects on neutrophils, were also run as 3 experiments on 3 different days (i.e., experiment 1, 2, and 3; **Supplementary Figure 2B**). Here, each experiment consisted of CM from MDECs from one cow (i.e., MDEC Line 1) tested against neutrophils isolated from the blood of 3 individual cows (e.g., neutrophil j, k & l for experiment 1; **Supplementary Figure 2B**). For all assays, except those to determine ratios of intracellular/extracellular DNA, data within each experiment were averaged to generate data points on graphs and data points were analyzed by ANOVA. Each ANOVA was followed by a Dunnett’s multiple comparisons test. For assays used to determine intracellular/extracellular DNA at 9 h, Student’s t-tests were used to compare the means of the groups.

RNA deep sequencing was performed on neutrophils isolated from 3 cows, each treated with CM from MDEC Line 1 and control medium.

GraphPad Prism (GraphPad Software) was used to analyze all data processed by the authors. RNAseq data was analyzed by the Cornell University Biotechnology Resource Center, as described above. *P* < 0.05 was considered significant and P-values for each comparison are both indicated in the manuscript text and on the graphs.

## Results

### Bovine mammosphere-derived epithelial cell conditioned medium does not reduce neutrophil viability

Immediately after isolation, neutrophils were stained with trypan blue and live cells were counted using a light microscope (VWR, Radnor, PA). Based on the exclusion of trypan blue dye, nearly 100% neutrophils were viable (data not shown). To determine if MDEC CM is toxic to neutrophils in the planned functional experiments, neutrophils were incubated with RPMI + 2% FBS (control), MDEC CM from 3 individual cell lines, or busulfan (positive control) for 1 h, and maintained for 5 h in RPMI + 2% FBS before being analyzed for dead cells and activated caspases associated with apoptosis, mimicking the timeline used in the functional assays. No differences in viability between neutrophils incubated in RPMI + 2% FBS and those incubated in MDEC CM from cell lines 1 (p = 0.1845), 2 (p = 0.0547), and 3 (p = 0.0530), were observed and the average viability of neutrophils incubated in MDEC CM was > 90% (**Supplementary Figure 3A**). In contrast, busulfan treatment did significantly reduce neutrophil viability when compared to the RPMI + 2% FBS control (p < 0.0001) (**Supplementary Figure 3A**). Caspase activity, as a measure of apoptosis, in live neutrophils incubated in MDEC CM from the 3 individual cell lines did not increase when compared to the RPMI + 2% FBS control (p = 0.8923, p = 0.8921 and p = 0.9534, respectively), while caspase activity in live neutrophils treated with busulfan significantly increased (p = 0.0216) [**Supplementary Figure 3B** (i)]. To assure evidence of caspase activity was not missed by excluding dead cells from the analysis, data were reanalyzed taking all neutrophils (live and dead) into consideration. Again, neutrophils incubated in MDEC CM from the 3 individual cell lines did not exhibit elevated active caspase activity when compared to the RPMI + 2% FBS control (p = 0.2904, p = 0.1331 and p = 0.1918, respectively) [**Supplementary Figure 3B** (ii)], whereas neutrophils treated with busulfan showed significantly more active caspase activity compared to those incubated with RPMI + 2% FBS control medium (p < 0.0001) [**Supplementary Figure 3B** (ii)]. These data show that MDEC CM does not reduce neutrophil viability or induces apoptosis after 6 h of culture.

### Bovine mammosphere-derived epithelial cell conditioned medium stimulates neutrophil chemotaxis but does not significantly alter phagocytosis

**Figure 1:**
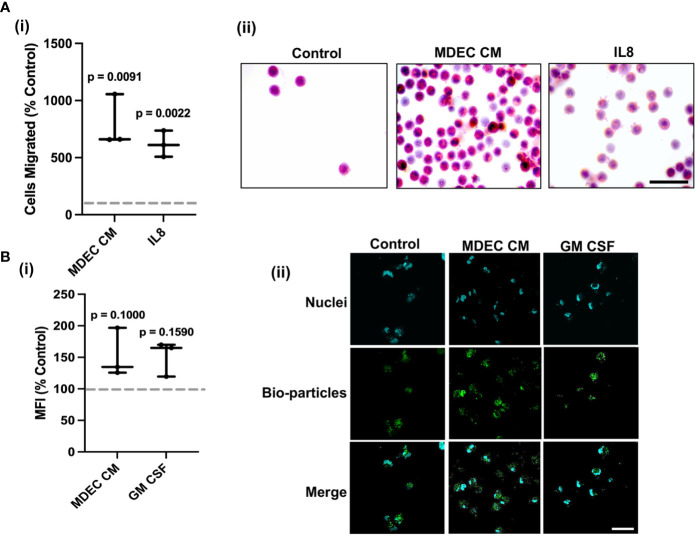
Bovine mammosphere-derived epithelial cell (MDEC) conditioned medium (CM) stimulates neutrophil chemotaxis and does not significantly increase phagocytosis. **(A)**. Bovine neutrophil chemotaxis was measured by counting cells that migrated through a mesh transwell insert into either RPMI control medium, MDEC CM, or medium containing the chemoattractant interleukin-8 (IL8). Cells migrated, expressed as percent control are shown (i), as are representative brightfield images of migrated cells stained with hematoxylin and eosin (ii). **(B)**. Neutrophil phagocytosis was determined by incubating cells with either RPMI control medium, MDEC CM, or medium containing granulocyte-macrophage colony-stimulating factor (GM CSF). Labeled *E. coli* bioparticles were added and intracellular particles were detected by flow cytometry. Mean fluorescent intensities (MFI) expressed as percent control are shown (i), as are representative fluorescent images (ii). Phagocytized particles are green, the nuclear stain 4’6-diamidino-2-phenylindole (DAPI) is blue. Each data point on graphs represents the results from one experiment, assessing the effects of CM from one MDEC line on 3 neutrophil preparations. Gray dotted lines on graphs indicate the control conditions expressed as 100%. n = 3 experiments. Scale bars = 50 µm.

Bovine MDEC CM collected from Cell Line 1 stimulated neutrophil migration in a chemotaxis assay (p = 0.0091). As expected, recombinant bovine interleukin-8 (IL8), a known chemotactic factor for bovine neutrophils (62), did as well (p = 0.0022). The effect of each was statistically significant as compared to the RPMI medium control (dotted line) [**Figure 1A** (i)]. Representative images of hematoxylin and eosin-stained neutrophils that migrated out of the transwell inserts they were plated in are shown in **Figure 1A** (ii). Bovine MDEC CM collected from Cell Line 1 did not significantly increase neutrophil phagocytosis as compared to the RMPI medium control (dotted line) (p = 0.100). The positive control bovine granulocyte-macrophage colony-stimulating factor (GM CSF), a cytokine reported to increase the phagocytic activity of bovine neutrophils (63) did not either (p = 0.159 [**Figure 1B** (i)]. Representative images of neutrophils showing phagocytosed bio-particles (green) and nuclei (blue) are shown in **Figure 1B** (ii).

To confirm that the effects of the MDEC CM on neutrophils were not specific to CM collected from Cell Line 1, chemotaxis and phagocytosis assays were repeated with CM generated from MDEC lines isolated from two additional cows (i.e., MDEC Lines 2 & 3). The overall patterns of these CM on neutrophil chemotaxis and phagocytosis were like those with CM from Cell Line 1 (**Supplementary Figures 4A, B**).

### Bovine mammosphere-derived epithelial cell conditioned medium suppresses reactive oxygen species accumulation and the expression of ROS-related proteins in neutrophils

**Figure 2:**
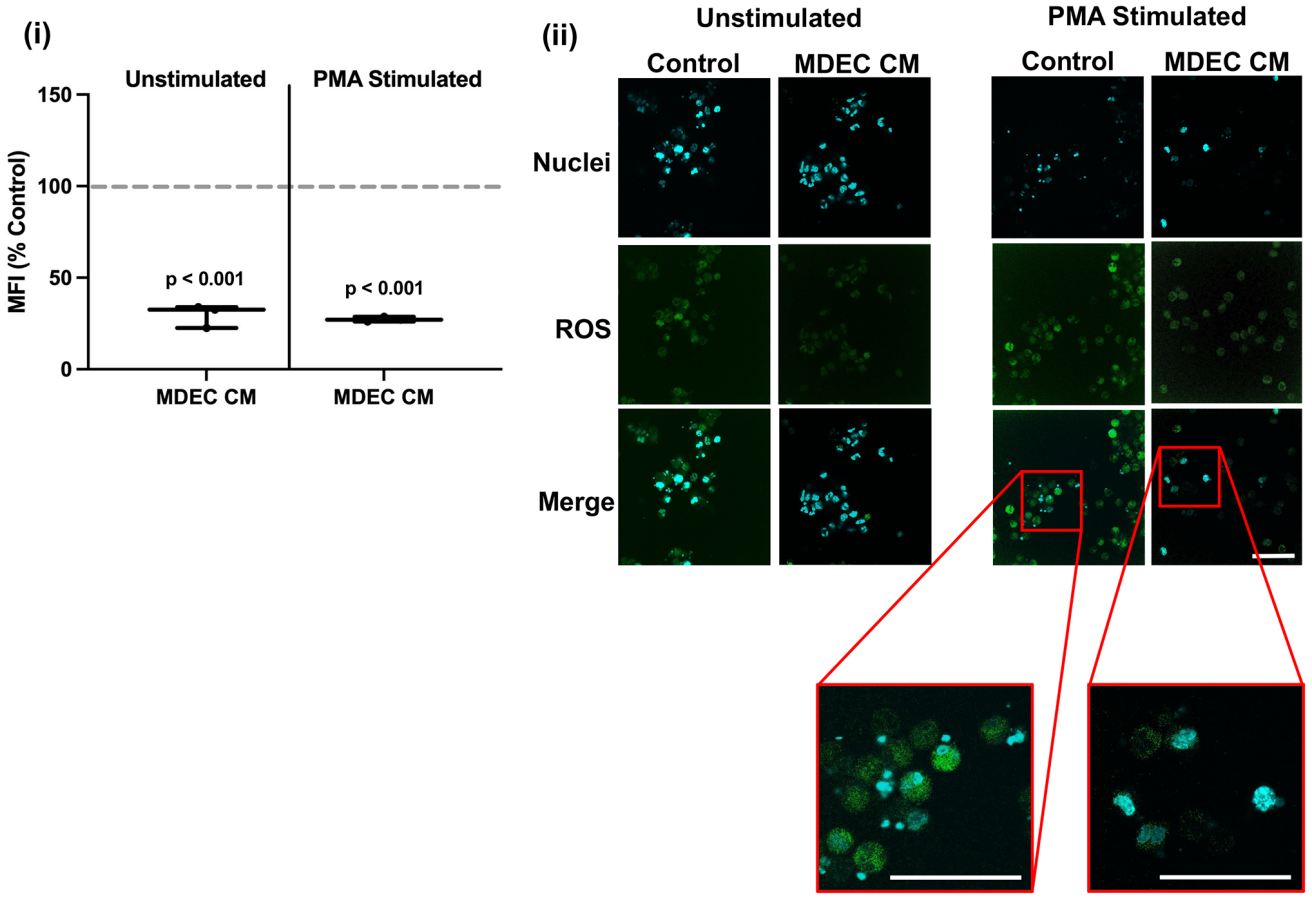
Bovine mammosphere-derived epithelial cell (MDEC) conditioned medium (CM) reduces the accumulation of reactive oxygen species (ROS) in neutrophils. ROS accumulation was quantified in neutrophils incubated in either RPMI control medium or MDEC CM, plus or minus the stimulant phorbol myristate acetate (PMA). 2’,7’-Diochlorodihydrofluorescein diacetate (H2DCFDA) was added to the cultures and the intracellular oxidized form was measured by flow cytometry. Mean fluorescent intensities (MFI) expressed as percent control are shown (i), as are representative fluorescent images (ii). ROS are indicated by green fluorescence, the nuclear stain 4’6-diamidino-2-phenylindole (DAPI) is blue. High magnification fluorescent images (red boxes) of nuclei in cultures treated with RPMI control medium or MDEC CM and stimulated with PMA, show that nuclear fragmentation characteristic of PMA treatment is lacking in cells treated with MDEC CM. Each data point on graphs represents the results from one experiment, assessing the effects of CM from one MDEC line on 3 neutrophil preparations. Gray dotted lines on graph indicate the control conditions expressed as 100%. n = 3 experiments. Scale bars = 50 µm.

In contrast to the stimulatory effect of MDEC CM on neutrophil chemotaxis, CM collected from MDEC Line 1 significantly inhibited neutrophil ROS accumulation in both unstimulated neutrophils (p < 0.001) and neutrophils stimulated with phorbol myristate acetate (PMA), a protein kinase C agonist that leads to the release of superoxide anions (64, 65) (p < 0.001), when compared to RPMI medium control (dotted line) [**Figure 2** (i)]. In addition to showing less ROS accumulation (green), fragmented neutrophil nuclei (blue) in response to PMA stimulation also appeared to be reduced by MDEC CM treatment [**Figure 2** (ii) inserts designated by red boxes], although this was not formally quantified. We confirmed that the inhibitory effect on neutrophil ROS is a general feature of MDEC CM by repeating the experiments with CM generated from MDEC Lines 2 & 3 (**Supplementary Figure 4C**).

PMA-stimulated ROS accumulation by bovine neutrophils is known to lead to the release of neutrophil extracellular traps (NETs) (66), web-like structures comprised of chromatin and chromatin-associated proteins that trap pathogens and limit infection, but also damage host tissue and lead to delayed tissue repair (67). In order to quantify the effects of MDEC CM on NET production, we used a commercially available enzyme activity assay to measure neutrophil elastase (NE), a secreted serine protease that localizes to NETs (68), in culture media. There was no difference in secreted NE detected in the medium of unstimulated neutrophils cultured in the presence of MDEC CM from MDEC Cell Line 1 when compared to neutrophils cultures in control medium (p = 0.2029). Significantly less NE was detected in culture medium collected from PMA-stimulated neutrophils cultured in the presence of MDEC CM from MDEC Cell Line 1 when compared to those cultured in control medium (p = 0.0011) [**Supplementary Figure 5A**(i)]. In human neutrophils, PMA stimulates NE secretion, while in bovine neutrophils, PMA may stimulate expression, but not secretion, of NE (69). Our results support the latter, as NE concentrations in all bovine neutrophil culture media were at the low end of the standard curve used to quantify NE in test samples [**Supplementary Figure 5A** (ii)].

**Figure 3:**
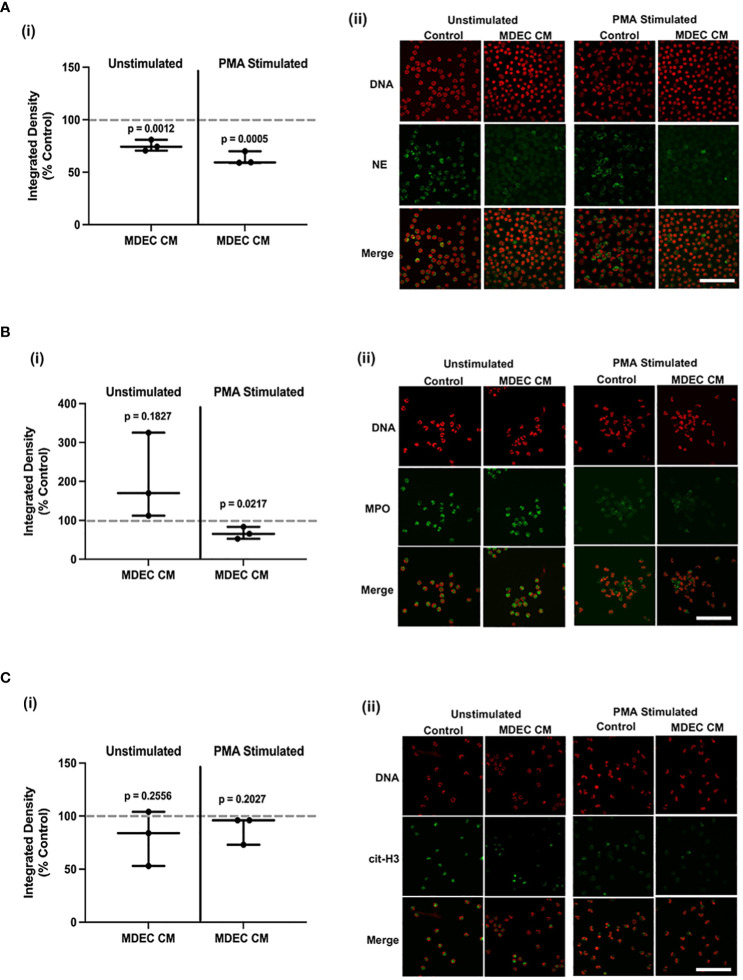
Bovine mammosphere-derived epithelial cell (MDEC) conditioned medium (CM) suppresses expression of proteins associated with reactive oxygen species (ROS) and neutrophil extracellular traps (NETs). **(A–C)**. Quantification (i) and representative images (ii) of neutrophil elastase (NE) **(A)**, myeloperoxidase (MPO) **(B)**, and citrullinated histone 3 (H3) **(C)** expression in bovine neutrophils incubated in either RPMI control medium or MDEC CM, plus or minus the stimulant phorbol myristate acetate (PMA). NE, MPO and cit-H3 are labeled with a green fluorophore, SYTOX^™^ orange labels DNA. Each data point on graphs represents the results from one experiment, assessing the effects of CM from one MDEC line on 3 neutrophil preparations. Gray dotted lines on graphs indicate the control conditions expressed as 100%. n = 3 experiments. Scale bars = 100 µm.

To robustly examine the expression of NET-associated proteins in bovine neutrophils, we used antibodies against NE, myeloperoxidase (MPO), and citrullinated histone H3 (cit-H3), on unstimulated and PMA-stimulated neutrophils cultured in the presence of MDEC CM from MDEC Cell Line 1 and unstimulated and PMA-stimulated neutrophils cultured in control medium. Analysis of NE antibody binding revealed a lower expression of NE in unstimulated (p = 0.0012) and PMA-stimulated (p = 0.0005) bovine neutrophils cultured in the presence of MDEC CM from MDEC Cell Line 1 when compared to those cultured in control media. [**Figure 3A** (i)]. The expression of MPO did not differ in unstimulated neutrophils cultured in MDEC CM as compared to control medium (p = 0.1827), but MDEC CM did reduce MPO expression in PMA-stimulated neutrophils (p = 0.0217) [**Figure 3B** (i)]. MDEC CM did not affect cit-H3 antibody binding in unstimulated or PMA-stimulated neutrophils as compared to the control media (p = 0.2256 and p = 0.2027 respectively) [**Figure 3C** (i)]. Representative images of NE, MPO and cit-H3 antibody binding are shown in **Figure 3** (ii) panels with antibody binding sites labeled in green and DNA labeled in orange. Images of neutrophils probed with mouse and rabbit isotype controls revealed little to no non-specific antibody binding based on the lack of green fluorescence (**Supplementary Figure 5B**).

### Mammosphere-derived epithelial cell conditioned medium changes the ratio of bovine neutrophil intracellular/extracellular DNA over time

**Figure 4:**
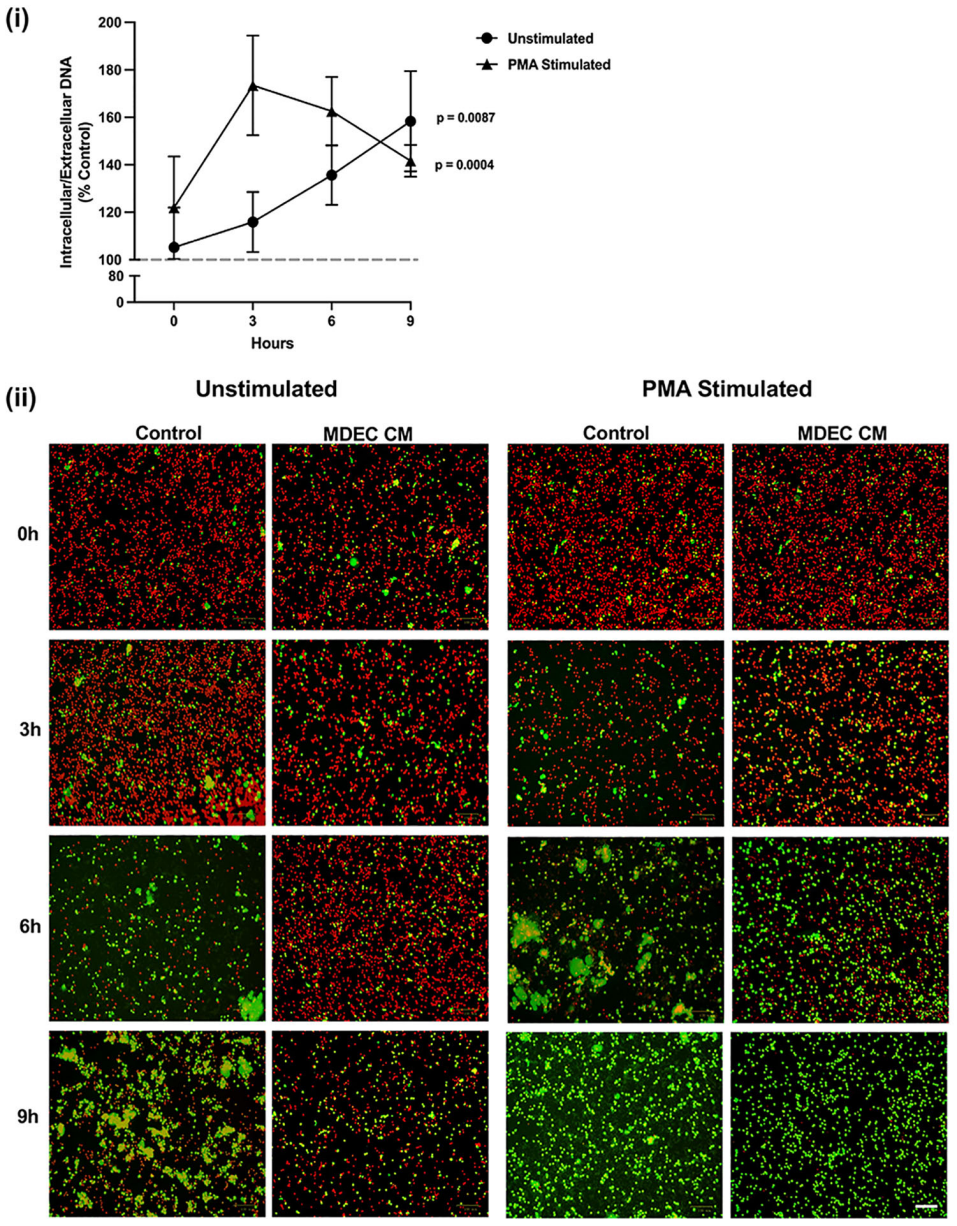
Neutrophils exposed to mammosphere-derived epithelial cell (MDEC) conditioned medium (CM) exhibit an increased ratio of intra- to extracellular DNA. After a 1 h treatment with MDEC CM, neutrophils were exposed to a nuclear red DNA labeling reagent (intracellular) and an extracellular green DNA labeling reagent (extracellular). Cells were imaged at 0, 3, 6 and 9 h post treatment and the ratio of intra- to extracellular DNA at each timepoint was calculated (i). Representative fluorescent images (ii). Each data point on graphs represents the average results from 3 experiments, each assessing the effects of CM from one MDEC line on 3 neutrophil preparations. Gray dotted line on graph indicates the control conditions expressed as 100%. n = 3 experiments. Scale bar = 100 µm.

As another way of exploring the effects of MDEC CM on neutrophil NET production, we evaluated overall intracellular (red) versus extracellular (green) DNA in unstimulated and PMA-stimulated neutrophils at multiple time points, using location-specific DNA dyes. Both unstimulated (p < 0.0087) and PMA-stimulated (p = 0.0004) neutrophils cultured in MDEC CM exhibited a higher ratio of intra- to extracellular DNA when compared to neutrophils cultured in control medium after 9 h of treatment [**Figure 4** (i)]. Images taken at 0, 3, 6 and 9 h capture the progression and patterns of intracellular/extracellular DNA in cultures over time [**Figure 4** (ii)]. As extracellular DNA is not NET-specific but also may be a product of cell death, we cannot conclude from these data that all green staining represents NETs. However, since the viability of unstimulated neutrophils incubated in control medium or MDEC CM was high after 6 h in culture (**Supplementary Figure 3A**) we propose that most of the green staining does represent NETs.

After determining that MDEC CM exerts functional effects on neutrophils, we took a two-pronged approach to determine how CM interacts with these innate immune cells. First, to define the neutrophil molecular pathways triggered by MDEC CM, we used an ELISA and fluorescent bead-based multiplex assay to measure cytokines secreted by neutrophils treated with MDEC CM. When these assays did not provide us with clear information (described in next paragraph), we followed up with RNA deep sequencing (RNAseq) to uncover transcriptional changes in neutrophils treated with MDEC CM. To approach the interaction of MDEC CM with neutrophils from another angle, we determined which MDEC secreted factors are responsible for the functional effects on neutrophils by analyzing and manipulating the MDEC CM.

### Neutrophil cytokine secretion is affected inconsistently in response to mammosphere-derived epithelial cell conditioned medium treatment

To define which molecular pathways in neutrophils are influenced by MDEC CM, we used an enzyme-linked immunosorbent assay (ELISA) to measure C-X-C motif chemokine 6 (CXCL6) and a fluorescent bead-based multiplex assay to measure interleukin-10 (IL10), tumor necrosis factor alpha (TNFα), and interferon gamma (IFNγ), in medium collected from neutrophils after MDEC CM treatment. RPMI + 2% FBS was included as a control medium to distinguish cytokines produced by neutrophils from those present in the CM used as treatment. IFNγ was not detected in any of the samples. Media collected from neutrophils treated with MDEC CM contained variable levels of CXCL6, IL10 and TNFα as compared to control media collected from neutrophils (**Supplementary Figure 6A**, **Supplementary Table 3**). Concentrations of the anti-inflammatory cytokine IL10 were all higher in media from neutrophils treated with MDEC CM when compared to media from neutrophils treated with the control medium, with CM from 2 out of the 3 MDEC lines resulting in a significantly higher production (p = 0.0087, p = 0.0232, p = 0.1654). The concentrations of the inflammatory cytokines CXCL6 and TNFα, on the other hand, were highly variable across samples, so no general conclusions about the impacts of MDEC CM on neutrophil inflammatory cytokine production could be made (**Supplementary Figure 6A**, **Supplementary Table 3**).

### Global changes in neutrophil gene expression in response to mammosphere-derived epithelial cell conditioned medium are masked by neutrophil cow-of-origin effect

**Figure 5:**
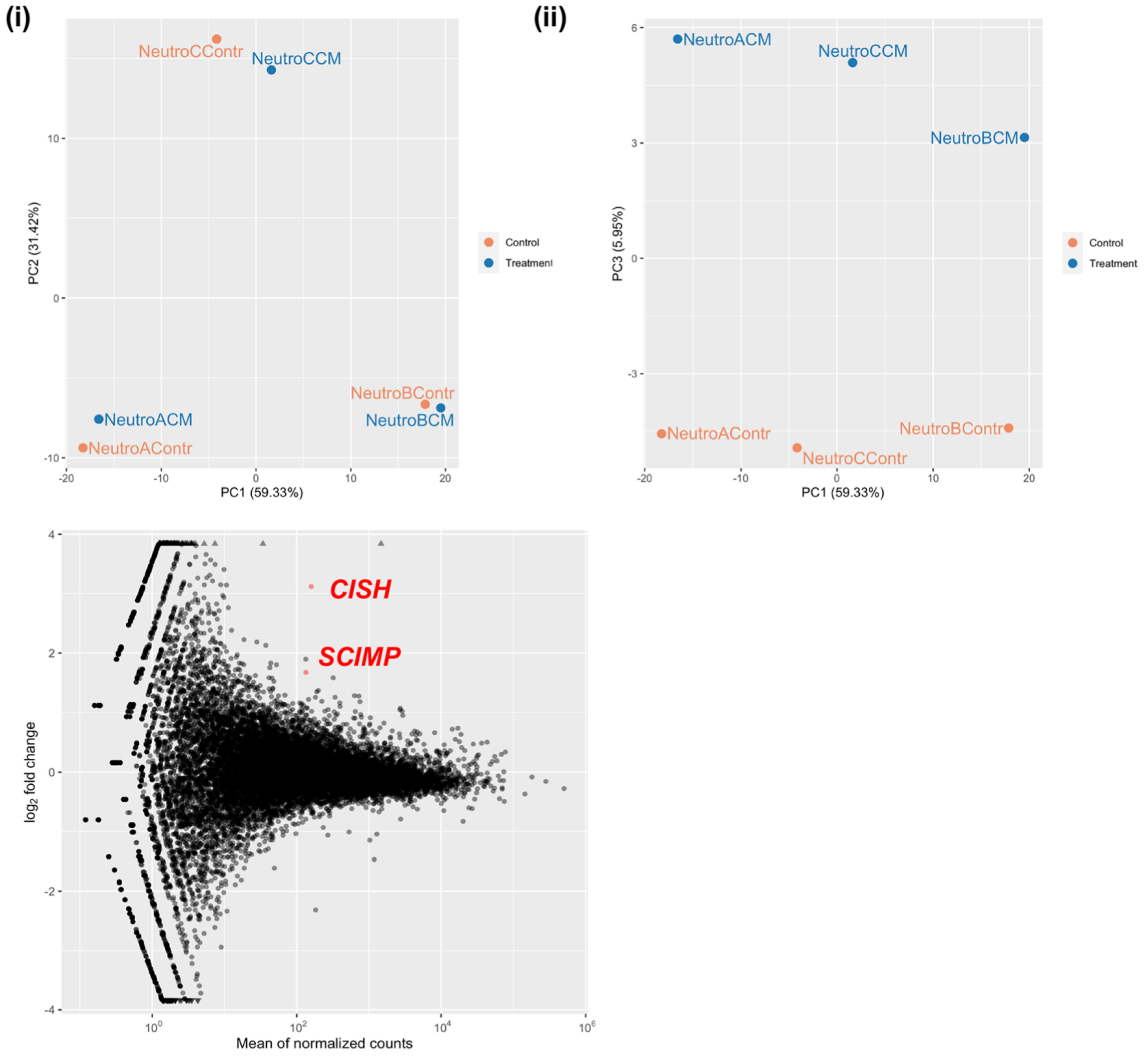
Global changes in neutrophil gene expression in response to mammosphere-derived epithelial cell (MDEC) conditioned medium (CM) are masked by cow-of-origin effect. Neutrophils from 3 individual cows were incubated with either MDEC CM or control medium before mRNA was isolated and sequenced. **(A)**. Cow-of-origin effect (PC1) (i) overwhelmed treatment effect (PC3) (ii). **(B)**. Only 2 differentially expressed genes (DEG) were detected across treatments. *CISH:* cytokine inducible SH2-containing protein, *SCIMP:* CSK interacting membrane protein.

As we were not successful at predicting the specific molecular pathways responsible for the functional effects of MDEC CM on neutrophils by evaluating changes in neutrophil cytokine production, we performed RNA deep sequencing (RNAseq) on neutrophils isolated from 3 individual cows to identify global transcriptional changes induced by MDEC CM treatment. Each neutrophil culture was incubated with MDEC CM from Cell Line 1 or control medium prior to mRNA extraction and sequencing. Principle component (PC) analysis of the sequencing data showed that cow-of-origin (PC1, PC2) effect was stronger than treatment (PC3) effect [**Figure 5A** (i, ii)], resulting in only 2 genes that were differentially expressed between treatment groups. Both genes were upregulated in neutrophils treated with MDEC CM when compared to control medium treatment (**Figure 5B**; **Supplementary Table 4**). The two upregulated genes were cytokine inducible SH2-containing protein *(CISH)* and CSK interacting membrane protein *(SCIMP)* (p-adj = 6.8x10^-08^ and p-adj = 0.0458, respectively). CISH is a cytokine inducible protein expressed in hematopoietic cells that suppresses cytokine signaling, thereby negatively regulating granulocyte production and growth (70, 71), and SCIMP is a transmembrane adaptor that promotes Toll-like receptor 4 (TLR4)-modulated proinflammatory cytokine responses (72, 73). As these 2 genes were detected as differentially expressed between MDEC CM- and control medium-treated neutrophils despite the strong influence of neutrophil cow-of-origin effect, these data are robust. However, the overwhelming impact of cow-of-origin did mask any additional data we anticipated to acquire with this experiment and thus, we were unable to identify specific molecular pathway alterations in MDEC CM-treated neutrophils.

### Bovine mammosphere-derived epithelial cell conditioned medium contains proteins known to affect neutrophil function

To identify bioactive factors in bovine MDEC CM that could be responsible for functional effects on neutrophil chemotaxis, phagocytosis, and ROS production, we searched a data set previously generated in our lab that globally characterized bovine MDEC CM using liquid chromatography-mass spectrometry (23). This analysis resulted in the identification of 347 proteins (23) and of these, we identified 11 proteins that have been reported to affect neutrophil functions (74–86) (**Table 3**). Of those 11 proteins, we were able to use enzyme linked immunosorbent assays (ELISAs) to detect and quantify the concentrations of 7 proteins in MDEC CM; namely complement components C3 (C3) and C5a (C5a), bovine CXC motif chemokine ligand 6 (CXCL6), insulin like growth factor 2 (IGF)2, superoxide dismutase 1 (SOD), peroxiredoxin 2 (PII) and catalase (CAT) (**Supplementary Table 5**). We selected 5 of these proteins (CXCL6, IGF2, SOD, PII and CAT) for functional follow up to determine if they indeed mediate the effects of MDEC CM on neutrophil chemotaxis, phagocytosis, and ROS production (**Table 3**). Although we did detect C3 and C5a in MDEC CM, we decided to exclude them for functional follow-up, as substantial concentrations were also detected in control medium (**Supplementary Table 5**), complicating the separation of complement component activity from MDEC CM from complement component activity from the base control culture medium.

**Table 3:** Proteins in bovine mammosphere-derived epithelial cell (MDEC) conditioned medium (CM) affecting neutrophil functions, as detected by liquid chromatography-mass spectrometry.

Protein Name	Abbreviation	Function	References
complement C3	C3	activates neutrophils	(74)
complement C5a	C5a	activates neutrophils	(75)
C-X-C motif chemokine 16	CXCL16	neutrophil chemoattractant	(76)
**C-X-C motif chemokine 6** ^a^	**CXCL6**	neutrophil chemoattractant	(77, 78)
protein S100-A11	S100-A11	stimulates neutrophil IL-6 and TNF secretion	(79)
**insulin-like growth factor II**	**IGF2**	enhances phagocytosis by neutrophils	(80)
pro-transforming growth factor	pro-TGF	polarizes neutrophils	(81)
**superoxide dismutase**	**SOD**	reduces superoxide radicals	(82, 83)
**peroxiredoxin-2**	**PII**	reduces peroxides	(84, 85)
peroxiredoxin-5	PV	reduces peroxides	(84, 85)
**catalase**	**CAT**	protects from oxidative damage	(86)

a Proteins in bold font were selected for functional follow up.

### Reducing chemokine (C-X-C motif) ligand 6 in mammosphere-derived epithelial cell conditioned medium decreases neutrophil chemotaxis but reducing insulin-like growth factor 2 has no effect on phagocytosis

**Figure 6:**
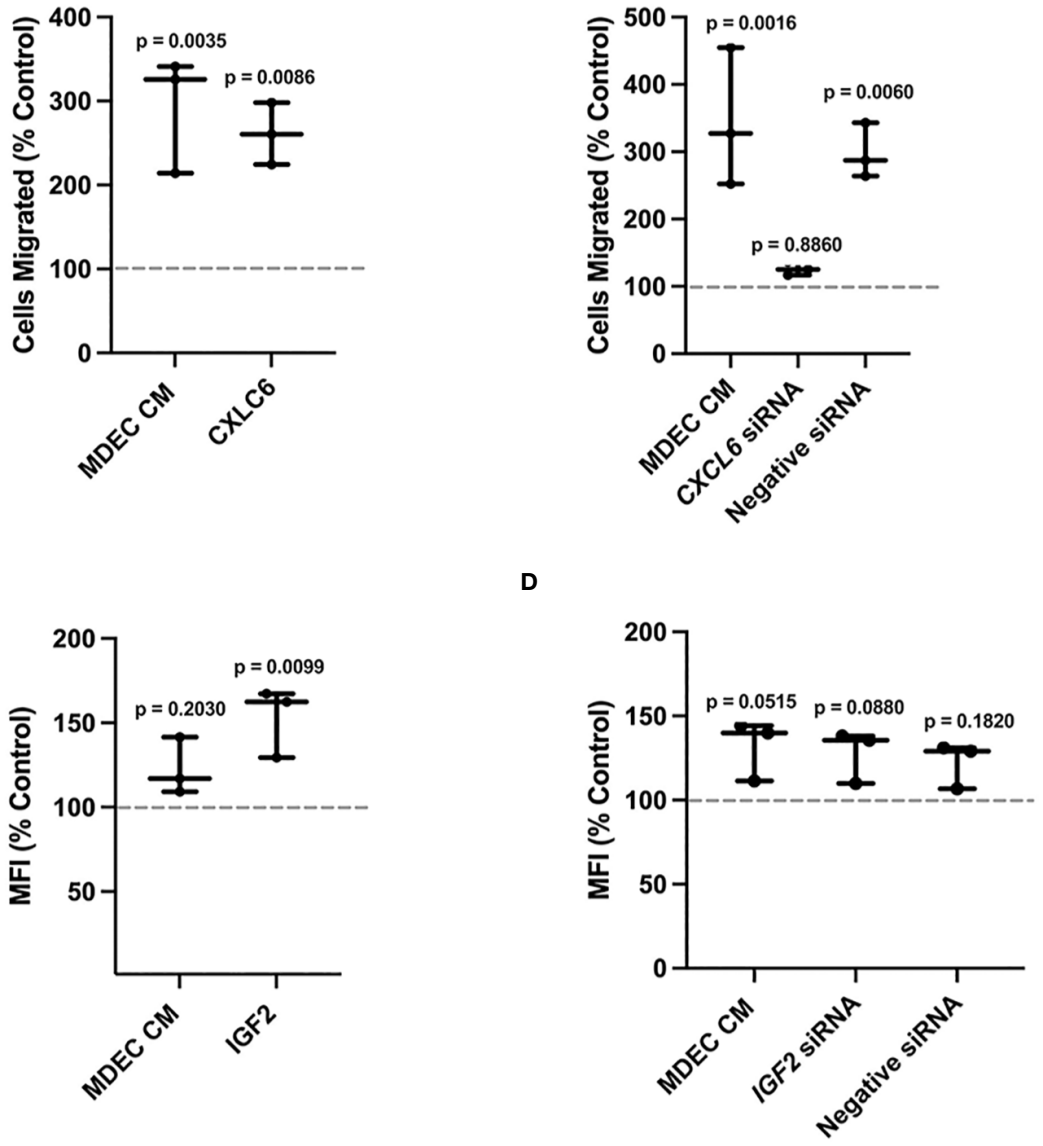
Reducing Chemokine (C-X-C motif) ligand 6 (CXCL6) expression in bovine mammosphere-derived epithelial cells (MDECs) changes the effect of conditioned medium (CM) on neutrophil chemotaxis, while reducing insulin-like growth factor 2 (IGF2) expression in MDECs does not change the effect of conditioned medium (CM) on neutrophil phagocytosis. **(A)**. Recombinant bovine CXCL6 was added to bovine neutrophils to determine if chemotaxis changed relative to RPMI control medium (dotted line). Cells treated with MDEC CM were included as a control. **(B)**. *CXCL6* siRNA was used to knock down CXCL6 in MDEC CM. Chemotaxis was assessed in neutrophils treated with RPMI control medium, MDEC CM, MDEC CM from cells transfected with *CXCL6* siRNA, and MDEC CM from cells transfected with a non-specific (negative) siRNA. **(C)**. Recombinant bovine IGF2 was added to bovine neutrophils to determine if phagocytosis changed relative to the RPMI control (dotted line). Cells treated with MDEC CM were included as a control. **(D)**. *IGF2* siRNA was used to knock down IGF2 in MDEC CM. Phagocytosis was assessed in neutrophils treated with RPMI control medium, MDEC CM, MDEC CM from cells transfected with *IGF2* siRNA and MDEC CM from cells that received a non-specific (negative) siRNA. Each data point on graphs represents the results from one experiment, assessing the effects of CM from one MDEC line on 3 neutrophil preparations. Gray dotted lines on graphs indicate the control conditions expressed as 100%. n = 3 experiments.

To evaluate whether CXCL6 in MDEC CM is responsible for the stimulating effect on neutrophil migration, we first repeated the chemotaxis assays with MDEC CM or recombinant bovine CXCL6 as chemoattractants. We found that MDEC CM and recombinant CXCL6 each stimulated neutrophil migration (p = 0.0035, p = 0.0086 respectively), and that these increases were statistically significant when compared to the RPMI medium control (dotted line) (**Figure 6A**). Next, we reduced CXCL6 in MDEC CM by silencing *CXCL6* expression in bovine MDECs using RNA interference. We confirmed knockdown of CXCL6 by a *CXCL6*-specific, but not a negative, small-interfering RNA (siRNA) on both mRNA and protein levels [**Supplementary Figures 6A, B** (i, ii)]. We then repeated the chemotaxis assays using MDEC CM, CM collected from MDECs transfected with *CXCL6* siRNA, or CM collected from MDECs transfected with a non-specific (negative) siRNA and compared neutrophil migration in each type of CM to that in control medium. As expected, complete CM and CM from MDECs transfected with the negative siRNA stimulated neutrophil migration significantly when compared to control medium (dotted line) (p = 0.0016, p = 0.0060 respectively) (**Figure 6B**). In contrast, neutrophil migration in the presence of CM from MDECs transfected with the *CXCL6* siRNA, thus with reduced CXCL6, was not significantly different from migration in the presence of control medium (p = 0.8860), indicating that CXCL6 in CM promotes neutrophil chemotaxis (**Figure 6B**).

We next used a similar approach, i.e., using a recombinant bovine protein and RNA interference, to determine whether IGF2 in MDEC CM is responsible for stimulating effect on neutrophil phagocytosis. MDEC CM did not lead to a significant increase in neutrophil phagocytosis (p = 0.2030) (**Figure 6C**). Use of recombinant bovine IGF2 did stimulate neutrophil phagocytosis, which was statistically significant when compared to control medium (p = 0.0099) (**Figure 6C**). Follow up experiments consisting of transfecting MDECs with an IGF2 specific siRNA, which decreased *IGF2* gene expression in MDECs and IGF2 protein in MDEC CM [**Supplementary Figures 6C, D** (i, ii)], showed that MDEC CM with decreased IGF2 did not lead to an increase in phagocytosis when compared to control medium (p = 0.0880) (**Figure 6D**). This suggests that IGF2 in MDEC CM is not responsible for any stimulatory effect MDEC CM has on neutrophil phagocytosis, however, since the level to which complete MDEC CM affects neutrophil phagocytosis varied depending on the MDEC Cell Line used for CM (**Figure 1B**; **Supplementary Figure 4B**), no concrete conclusions can be drawn regarding the role IGF2 in MDEC CM on this process.

### Reducing superoxide dismutase, peroxiredoxin 2, and catalase, in bovine mammosphere-derived epithelial cell conditioned medium increases neutrophil reactive oxygen species accumulation

**Figure 7:**
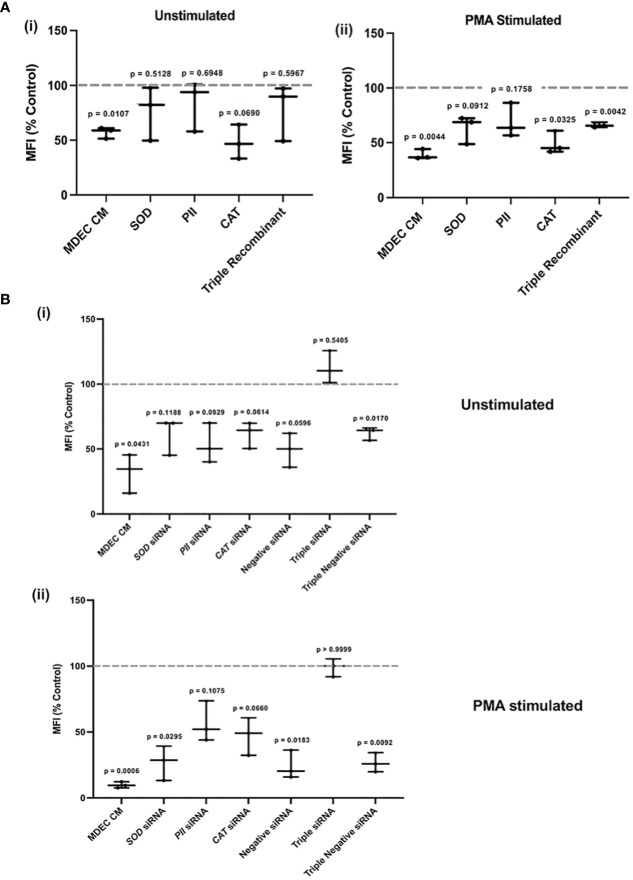
Reducing superoxide dismutase (SOD), peroxiredoxin 2 (PII), and catalase (CAT), expression, alone or in combination, in bovine mammosphere-derived epithelial cells (MDECs) variably effects how conditioned medium (CM) influences the accumulation of reactive oxygen species (ROS) in neutrophils. **(A)**. Recombinant SOD, PII, CAT or all three combined (Triple Recombinant), were added to bovine neutrophils to determine if levels of ROS changed relative to the RPMI control. Cells treated with MDEC CM were included as a control. Experiments were conducted with (i) and without (ii) phorbol myristate acetate (PMA) stimulation. **(B)**. siRNAs were used to knock down SOD, PII, CAT, or a combination of all three (triple siRNA) in MDECs. ROS were assessed in neutrophils treated with RPMI control medium, MDEC CM, MDEC CM from cells transfected with *SOD, PII, CAT* or all three siRNAs, and MDEC CM from cells that received non-specific siRNA at the same concentrations as specific siRNAs. Experiments were conducted with (i) and without (ii) PMA stimulation. Each data point on graphs represents the results from one experiment, assessing the effects of CM from one MDEC line on 3 neutrophil preparations. Gray dotted lines on graphs indicate the control conditions expressed as 100%. n = 3 experiments.

As described above, we first evaluated neutrophil ROS in the absence and presence of PMA in either MDEC CM or medium with the addition of recombinant SOD, PII, and CAT, alone or in combination (triple recombinant), using protein concentrations recommended in other studies (87, 88). As expected, unstimulated or PMA-stimulated neutrophils in MDEC CM showed a significant reduction of ROS when compared to those in the medium controls (p = 0.0107, p = 0.0044 respectively) [**Figure 7A** (i, ii)]. The addition of SOD, PII, CAT, alone or the 3 proteins combined, to unstimulated neutrophils did not affect ROS accumulation (p = 0.5128, p = 0.6948, p = 0.0690 and p = 0.5967, respectively) [**Figure 7A** (i)]. The addition of SOD or PII to PMA-stimulated neutrophils did not affect ROS (p = 0.0912 and p = 0.1758, respectively), while treatment with CAT or the combination of all 3 proteins led to a significant decrease (p = 0.0325 and p = 0.0042, respectively) [**Figure 7A** (ii)]. To reduce the expression of SOD, PII and CAT, alone or in combination, in MDEC CM, siRNAs targeting *SOD, PII* and *CAT* were used. The negative siRNA, which does not correspond to any bovine mRNA sequence, was included as a control at the same concentration as the specific siRNAs (**Supplementary Figures 7A–E**, **8A, B**).

ROS accumulation in unstimulated neutrophils was significantly decreased when cells were incubated with complete MDEC CM (p = 0.0431), or CM collected from MDEC transfected with a triple dose of negative siRNA (p = 0.0170) [**Figure 7B** (i)]. CM from MDECs transfected with *SOD, PII*, *CAT*, all 3 combined or the negative siRNA did not affect ROS levels in unstimulated neutrophils (p = 0.1188, p = 0.0929, p = 0.0614, p = 0.5405 and p = 0.596, respectively) [**Figure 7B** (i)]. These data suggest that the activity of each of these proteins in MDEC CM influences neutrophil ROS accumulation, but since the negative control did not perform consistently, concrete conclusions about the involvement of each single protein cannot be drawn. Still, the simultaneous reduction of all 3 proteins in MDEC CM did change the effect of CM on ROS accumulation [**Figure 7B** (i)], demonstrating that the combination of these bioactive factors does reduce ROS in unstimulated neutrophils.

PMA-stimulated neutrophils treated with MDEC CM, CM with reduced SOD from MDECs transfected with either concentration of negative siRNA deceased when compared to the control (p = 0.006, p = 0.0295, p = 0.0183 and p = 0.0092, respectively) (**Figure 7B** (ii)). In contrast, ROS in PMA-stimulated neutrophils did not change when cells were treated with CM with reduced PII or catalase alone, nor with all 3 proteins combined (p = 0.1075, p = 0.660 and p = 0.999, respectively) (**Figure 7B** (ii)), supporting the independent roles of PII and CAT in MDEC CM in reducing ROS accumulation in neutrophils.

## Discussion

This study used *in vitro* functional immunological assays to demonstrate that the secretome of bovine mammosphere-derived epithelial cells (MDECs), delivered as conditioned medium (CM), affects bovine neutrophil functions. Specifically, bovine MDEC CM reliably stimulated neutrophil chemotaxis, increased neutrophil phagocytosis in a donor-dependent manner, and consistently inhibited reactive oxygen species (ROS) production by neutrophils. With additional work on characterization and delivery, the MDEC secretome may represent a promising host-directed immunotherapy (HDT) for the treatment of bacterial infections.

A limitation of this work is that we only evaluated one concentration of MDEC CM. Although we are interested in the information a dose-response experiment could provide, we have not fully optimized the techniques to do so. We have done experiments in which we diluted the CM before using it to treat target cells. Although we have previously shown that bovine MDEC CM contains a wide range of secreted proteins (23), the concentrations of these proteins was too low to repeatably exert consistent functional effects on target cells when the CM was diluted (data not shown). Another strategy is to concentrate the CM to evaluate the effects of higher doses. We have concentrated the CM by lyophilization followed by reconstitution in volumes lower than the original CM volume. However, this resulted in a concentrated CM that proved toxic to target cells, as it has salt concentrations higher than those that are optimal for cell culture. Although we could dialyze the concentrated CM to reduce salt concentrations, we have not yet successfully prepared a concentrated CM that is both suitable for treating cultured cells and is biologically active.

Although we only tested MDEC CM at one concentration, we found that the chemokine (C-X-C motif) ligand 6 (CXCL6) secreted by bovine MDECs increases neutrophil chemotaxis *in vitro*. In humans, CXCL6 is secreted by macrophages and epithelial cells during inflammation to attract neutrophils to sites of infection (89, 90). Studies in cows have reported that (i) bovine monocyte-derived macrophage *CXCL6* expression is altered during *M. bovis* infection (91), (ii) expression of *CXCL6* in bovine mammary tissue is upregulated during *E. coli* mastitis (92), (iii) *CXCL6* is upregulated in bovine mammary epithelial cell cultures in response to *S. aureus*-derived lipoteichoic acid (93), (iv) both bovine polymorphonuclear cells (PMNs) and a mammary epithelial cell line express *CXCL6* and (v) recombinant human CXCL6 has weak chemotactic effects on bovine PMN (94). These data collectively indicate that CXCL6 is involved in the recruitment of bovine neutrophils to infection sites, and thus, suggests a universal role of epithelial cell secreted CXCL6 in inflammation. Although the bovine chemokine repertoire is overall very similar to that found in humans and mice, differences have been noted, so it is important to confirm chemokine-associated mechanisms in specific mammalian species of interest (95).

We also observed that selective bovine MDEC CM stimulates phagocytosis of bioparticles by bovine neutrophils, but that this effect was most likely not exclusively mediated by insulin-like growth factor 2 (IGF2). The addition of recombinant bovine IGF2 to neutrophil cultures stimulated phagocytosis, providing an indirect clue as to its functional properties, however, reducing this protein in MDEC CM did not reliably change the effect of MDEC CM on neutrophil phagocytosis. One explanation could be that although IGF2 can stimulate phagocytosis, its efficacy is dampened by the presence of other bioactive factors in the rich mixture of secretome components. For example, it has been described that in biofluids IGF-binding proteins regulate IGF availability (96). Alternatively, additional proteins in bovine MDEC CM could have stronger stimulating effect on neutrophil phagocytosis, which could promote neutrophil phagocytosis even in the absence of IGF2. Although our previous mass spec analysis of bovine MDEC CM identified IGF2 as a top candidate responsible for promoting neutrophil phagocytosis (23), additional proteins such as the complement proteins C3 and C5a and protransforming growth factor (**Table 3**) could have similar effects on bovine cells, and thus, could be explored in more depth in future studies. As described in the Results section, we did not follow up on the roles of C3 and C5a from MDEC CM on neutrophil phagocytosis in this study, because while we detected these proteins in the CM by enzyme linked immunosorbent assay (ELISA) we also detected them in the control medium, making it difficult to determine the source of complement activity in the *in vitro* model systems we used.

In addition to stimulating neutrophil functions, including chemotaxis and phagocytosis, bovine MDEC CM suppressed the accumulation of reactive oxygen species (ROS) in neutrophils through a mechanism that most likely involves a concerted action of the proteins superoxide dismutase (SOD), peroxiredoxin 2 (PII), and catalase (CAT) present in bovine MDEC CM. Although neutrophils need to produce ROS to kill pathogens, dampening ROS may be therapeutically beneficial in the context of a bacterial infection if treatment goals are to reduce inflammation that leads to acute disease in the short term as well as long term disease and permanent tissue damage over time.

ROS induce redox-dependent signaling cascades that are critical for the maintenance of successful organ and tissue development and normal physiology (97). ROS regulate vascular diameter, mediate oxygen sensing, influence skeletal muscle physiology, contribute to genomic stability, and mediate immune responses (98–102). However, an excess of ROS can contribute to the pathology of inflammatory diseases in many species, including cows and humans (97, 103, 104). In cows, a systemic excess of ROS may lead to a state called oxidative stress (OS) (105, 106), which threatens overall health by inducing a range of metabolic and/or hormonal imbalances, and has been associated with two impactful diseases that negatively impact the dairy industry, mastitis and metritis (107–109). In addition, OS may promote dysfunctional inflammation associated with decreased fertility and milk yield and increased metabolic stress. Metabolic stress is a risk factor for ketosis, fatty liver disease and placental retention, as well as mastitis (105). As these conditions and diseases lead to economic losses for the dairy industry, strategies to mitigate excessive ROS in cows have been a high management priority (106, 110). In humans, OS contributes to the development and pathology of many diseases including cancer, respiratory disease, and neurodegenerative disorders (97, 111). For example, ROS dysregulation is involved in cancer development by causing accumulation of oncogenic mutations, altering cell metabolism and promoting metastasis (112–115). ROS contribute to various cancer pathologies not only by affecting cancer cells, but by modulating the tumor microenvironment as well (116). Endothelial cells, which form blood vessels to deliver nutrients to tumor cells are triggered by OS (117, 118) and innate and adaptive immune cell activity, critical to cancer progression, is heavily influenced by ROS (119–121). Because dysregulated ROS universally contributes to tissue damage and disease, an HDT that can control ROS accumulation may be valuable in both veterinary and human medicine.

SOD and catalase are specific enzymatic antioxidants that reduce ROS in human skin, and the development of skin diseases such as contact dermatitis, acne vulgaris, and cancer, is associated with an abnormal reduction of these proteins (122). PII overexpression protects against neuronal cell death in an Alzheimer’s model, leading to the suggestion that it might be a viable pharmacological target for the treatment of this disease (123). Based on the observations that (i) excessive ROS promotes dysfunctional inflammation and (ii) enzymatic antioxidants can reduce the negative effects of that inflammation, MDEC CM containing these antioxidant proteins may well serve as a rich source of bioactive factors for HDT for inflammatory diseases of cattle and humans, including those initiated by pathogen infection.

Future clinical trials will determine whether the MDEC secretome acts on neutrophils during infection on an organismal level like what it does on peripheral blood-derived neutrophils *in vitro.* A bovine experimentally induced mastitis model would be ideal for these types of experiments for multiple reasons. In this model, features of infection may be altered based on the pathogen(s) introduced, allowing for the study of various host-pathogen dialogs that determine the nature of the immune response (124, 125). Non-invasive read outs such as somatic cell count and bacterial load in milk can be used to follow the course of infection/treatment in real time. Cows also exhibit traits that make them a relevant model for human medicine. Cows, like humans, live in less regulated environments than laboratory mice, the most used animal model for human medical studies. Cows are large animals, allowing for generous sample collections. Efforts are made to maintain genetic diversity of dairy cows when breeding to enhance desirable traits (126, 127). This is not the case for most laboratory mice, which are purposefully bred to reduce genetic variance (128). Finally, the genetic diversity in cows and humans impacts the host immune response to infection (129–132), making the cow a potentially more accurate model for the study of human HDT than the mouse.

The natural diversity amongst individual dairy cows was evident in this study. In the functional assays, we observed similar trends in neutrophil responses to CM from 3 different MDEC lines, but the scale of neutrophil migration differed depending on which MDEC line was used and the degree to which bioparticles were phagocytosed by MDEC CM-treated neutrophils when compared to control-treated neutrophils was variable as well. Based on this, we decided to present the functional data from this study in individual graphs according to MDEC line to accentuate the patterns of neutrophil responses to CM without masking the effects by combining values that are not to scale or that are variable based on CM source. Neutrophil cytokine secretion in response to treatment with MDEC CM greatly varied, most likely based on CM source and/or neutrophil cow-of-origin. When comparing global transcriptomic data from neutrophils isolated from 3 different cows, each treated with CM from the same MDEC cell line and control medium, neutrophil cow-of-origin effect was found to be stronger than treatment effect.

Based on the overall results of this study, we propose that the MDEC secretome administered therapeutically to cattle may increase neutrophil recruitment and pathogen phagocytosis while reducing ROS production. As a result, pathogens may be more readily cleared and local inflammation and tissue damage might be reduced. Meeting these goals of HDT has the potential to improve post bacterial infection outcomes as well as reduce the burden of drug-resistant bacteria on both physical and economic health across species.

## Data availability statement

The original contributions presented in the study are included in the article/**Supplementary Material**, further inquiries can be directed to the corresponding author.

## Ethics statement

The animal study was approved by Cornell Institutional Animal Care and Use Committee. The study was conducted in accordance with the local legislation and institutional requirements.

## Author contributions

RH: Writing – review & editing, Writing – original draft, Methodology, Investigation, Formal analysis, Data curation, Conceptualization. AS: Writing – review & editing, Writing – original draft, Investigation, Formal analysis, Data curation, Conceptualization. KO: Writing – review & editing, Data curation. LO: Writing – review & editing, Supervision. LH: Writing – review & editing, Supervision. DN: Writing – review & editing, Supervision, Formal analysis. GV: Writing – review & editing, Writing – original draft, Supervision, Resources, Project administration, Funding acquisition, Formal analysis, Conceptualization.

## Supplementary material

Supplementary Figure 1 Flow cytometry gating scheme and Western blot reference protein validation. **(A)**. Flow cytometry data were gated as depicted. First, all cells were visualized based on side scatter area (SSC-A) versus forward scatter area (FSC-A). Then, single cells were gated based on forward scatter height (FSC-H) forward scatter area (FSC-A). From the single cell population, neutrophils were gated based on side scatter area (SSC-A) versus forward scatter area (FSC-A). Live neutrophils were gated based on lack of PI staining detected in Comp-PE_CF594-A. Finally, the mean fluorescent intensity (MFI) of live neutrophils was determined based on detection in Comp-FITC_BB515_A. **(B, C)**. Anti-fibronectin and anti-β-actin antibodies were tested on Western blots of bovine MDEC CM undiluted, diluted 1:2, and diluted 1:4, for validation as reference proteins. Band densities relative to controls were graphed (i) and representative images of blots are shown (ii). Gray dotted lines on graphs indicate the control conditions expressed as 100%. Data points on graphs represents the results from one experiment, assessing the effects of CM from one MDEC line on 3 neutrophil preparations. n = 3 experiments.

Supplementary Figure 2 Schematic of experiments and presentation of data. **(A)**. The initial chemotaxis, phagocytosis, ROS production and NE activity assays were run as 3 experiments, each on a different day. For an experiment, MDEC CM was collected from 3 MDEC cell lines (1, 2 & 3) and tested against neutrophils isolated from the blood of 3 cows. Within an experiment, data from the 3 neutrophil isolations treated with CM from each MDEC cell line were averaged to create the data points shown on the cell line specific graphs presented in **Figures 1**, **2**; **Supplementary Figures 3**-**5**. **(B)**. Viability, active caspase detection assays, intra-versus extracellular DNA analysis, as well as RT-PCR, Western blots, chemotaxis, phagocytosis, and ROS production assays designed to determine which bioactive factors in MDEC CM were responsible for these functional effects on neutrophils were run as 3 experiments. Each experiment consisted of MDEC CM collected from one cell line, tested against neutrophils isolated from the blood of 3 cows. Data from the 3 neutrophil preparations used for each experiment were averaged to create the data points shown on the graphs presented in **Figures 4**, **6**, **7**; **Supplementary Figures 1**, **2**, **6**. Image generated by Biorender.

Supplementary Figure 3 Bovine mammosphere-derived epithelial cell (MDEC) conditioned medium (CM) does not induce neutrophil cell death or apoptosis. **(A)**. Viability of neutrophils 6 hours (h) post isolation as detected by propidium iodide (PI) staining. Neutrophils were incubated for 1 h in control medium, MDEC CM from 3 cell lines or busulfan immediately after isolation, then maintained in control medium for 5 h before flow cytometric analysis. **(B)**. Active caspase activity in neutrophils 6 h post isolation as detected by TF2-VAD-FMK Neutrophils were incubated for 1 h in control medium, MDEC CM from 3 cell lines or busulfan immediately after isolation, then incubated with TF2-VAD-FMK for 5 h before flow cytometric analysis. Analysis of live neutrophils (i). Analysis of all neutrophils (ii). Gray dotted lines on graphs indicate the control conditions expressed as 100%. Data points on graphs represents the results from one experiment, assessing the effects of CM from one MDEC line on 3 neutrophil preparations. n = 3 experiments.

Supplementary Figure 4 Treatment of neutrophils with conditioned medium (CM) collected from multiple bovine mammosphere-derived epithelial cell (MDEC) lines leads to similar patterns of chemotaxis, phagocytosis, and reactive oxygen species (ROS) accumulation. **(A)**. Bovine neutrophil chemotaxis was measured by counting cells that migrated through a mesh transwell insert into either RPMI control medium, CM from 2 different MDEC lines, or medium containing the chemoattractant interleukin-8 (IL8). Cells migrated, expressed as percent control are shown. **(B)**. Neutrophil phagocytosis was determined by incubating cells with either RPMI control medium, CM from 2 different MDEC lines, or medium containing granulocyte-macrophage colony-stimulating factor (GM CSF). Labeled *E. coli* bioparticles were added and intracellular particles were detected by flow cytometry. Mean fluorescent intensities (MFI) expressed as percent control are shown. **(C)**. Bovine neutrophil ROS production was quantified in neutrophils incubated in either RPMI control medium or CM from 2 different MDEC lines, plus or minus the stimulant phorbol myristate acetate (PMA). 2’,7’-Diochlorodihydrofluorescein diacetate (H2DCFDA) was added to the cultures and the intracellular oxidized form was measured by flow cytometry. Mean fluorescent intensities (MFI) expressed as percent control are shown. Each data point on graphs represents the results from one experiment, assessing the effects of CM from one MDEC line on 3 neutrophil preparations. Gray dotted lines on graphs indicate the control conditions expressed as 100%. n = 3 experiments.

Supplementary Figure 5 Bovine mammosphere-derived epithelial cell (MDEC) conditioned medium (CM) suppresses neutrophil elastase (NE) secretion. **(A)**. Neutrophils were incubated in either RPMI control medium or MDEC CM, plus or minus the stimulant phorbol myristate acetate (PMA), and secreted bovine NE was quantified via enzyme activity assays (i). Standards of known concentrations were included in the assay, and a curve was generated to quantify NE production in test conditions (ii). Each data point on graphs represents the results from one experiment, assessing the effects of CM from 3 MDEC lines on 3 neutrophil preparations. n = 3 experiments. **(B)**. Images of neutrophils labeled with mouse or rabbit IgG as isotype controls for immunofluorescence assays. Mouse and rabbit IgG are labeled with a green fluorophore, SYTOX^™^ orange labels DNA. Scale bars = 100 µm.

Supplementary Figure 6 Bovine mammosphere-derived epithelial cell (MDEC) conditioned medium (CM) variably influences neutrophil cytokine production and RNA-interference (RNAi) decreases chemokine (C-X-C motif) ligand 6 (CXCL6) or insulin-like growth factor 2 (IGF2) expression in bovine MDECs. **(A)**. An enzyme-linked immunosorbent assay (ELISA) or fluorescent bead-based multiplex assay was used to measure the cytokines C-X-C motif chemokine 6 (CXCL6), interleukin-10 (IL10), tumor necrosis factor alpha (TNFα), and interferon gamma (IFNγ), in medium from neutrophils treated with CM from 3 MDEC Lines or control medium consisting of RPMI + 2% FBS. **(B)**. Quantitative reverse transcription-polymerase chain reaction (qRT-PCR) was used to detect target transcript expression in MDECs transfected with either short interfering RNA (siRNA) against *CXCL6* or a non-specific (negative) siRNA. **(C)**. Western blots (WBs) were run to detect protein expression in MDEC CM collected from MDECs transfected with siRNA against *CXCL6* or a negative siRNA. Band density as percent untransfected cells (no treatment) was calculated (i), representative images are shown (ii). **(D)**. qRT-PCR was used to detect target transcript expression in bovine MDECs transfected with either siRNA against *IGF2* or a negative siRNA. **(E)**. WBs were run to detect protein expression in MDEC CM collected from MDECs transfected with either siRNA against *IGF2* or a negative siRNA. Band density as percent untransfected cells (no treatment) was calculated (i), representative images are shown (ii). Gray dotted lines on graphs indicate the control conditions expressed as 100%. n = 3 replicates.

Supplementary Figure 7 RNA-interference (RNAi) decreases superoxide dismutase (SOD), peroxiredoxin 2 (PII), or catalase (CAT) expression in bovine mammosphere-derived epithelial cells (MDECs). **(A)**. Quantitative reverse transcription-polymerase chain reaction (qRT-PCR) was used to detect target transcript expression in MDECs transfected with either short interfering RNA (siRNA) against *SOD* or a non-specific (negative) siRNA. **(B)**. Western blots (WBs) were run to detect protein expression in MDEC CM collected from MDECs transfected with siRNA against *SOD* or a negative siRNA. Band density as percent untransfected cells (no treatment) was calculated (i), representative images are shown (ii). **(C)**. qRT-PCR was used to detect target transcript expression in bovine MDECs transfected with either siRNA against *PII* or a negative siRNA. **(D)**. WBs were run to detect protein expression in MDEC CM collected from MDECs transfected with either siRNA against *PII* or a negative siRNA. Band density as percent untransfected cells (no treatment) was calculated (i), representative images are shown (ii). **(D)**. qRT-PCR was used to detect target transcript expression in bovine MDECs transfected with either siRNA against *CAT* or a negative siRNA. **(D)**. WBs were run to detect protein expression in MDEC CM collected from MDECs transfected with either siRNA against *CAT* or a negative siRNA. Band density as percent untransfected cells (no treatment) was calculated (i), representative images are shown (ii). Gray dotted lines on graphs indicate the control conditions expressed as 100%. n = 3 replicates.

Supplementary Figure 8 RNA-interference (RNAi) decreases superoxide dismutase (SOD), peroxiredoxin 2 (PII), and catalase (CAT) expression in bovine mammosphere-derived epithelial cells (MDECs). **(A)**. Quantitative reverse transcription-polymerase chain reaction (qRT-PCR) was used to detect target transcript expression in MDECs transfected with either short interfering RNA (siRNA) against *SOD, PII* and *CAT* or a non-specific (negative) siRNA at the same concentration. **(B)**. Western blots (WBs) were run to detect protein expression in MDEC CM collected from MDECs transfected with siRNA against *SOD, PII* and *CAT* or a negative siRNA at the same concentration. Band density as percent untransfected cells (no treatment) was calculated. Gray dotted lines on graphs indicate the control conditions expressed as 100%. n = 3 replicates.
