# Supplementary figures and images for: The mammosphere-derived epithelial cell secretome modulates neutrophil functions in the bovine model
Source: Front Immunol. 2024 Jun 27;15:1367432. doi: 10.3389/fimmu.2024.1367432 (PMC11236729; doi:10.3389/fimmu.2024.1367432)

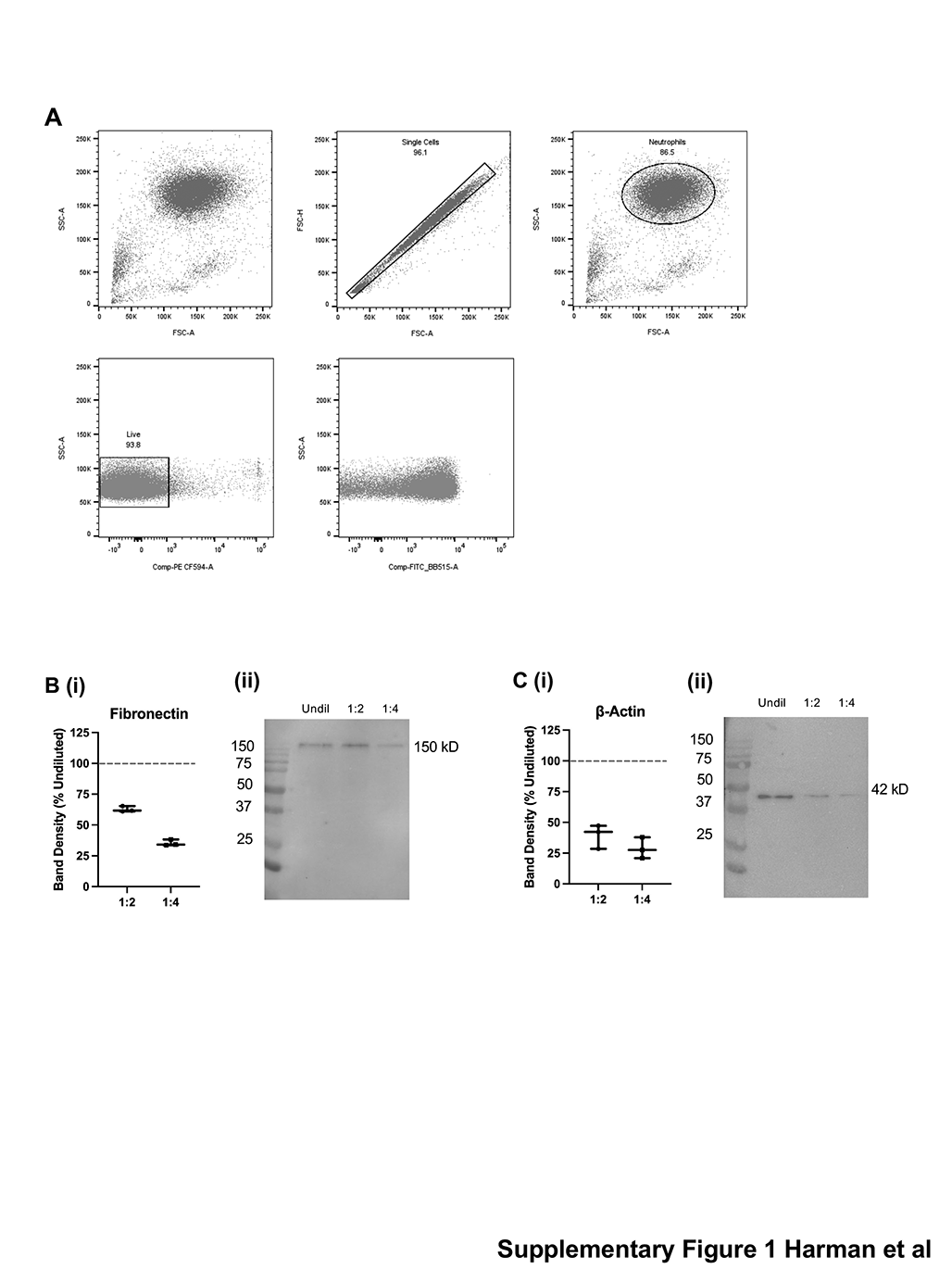

Supplement: Supplementary Figure 1 — Flow cytometry gating scheme and Western blot reference protein validation. (A). Flow cytometry data were gated as depicted. First, all cells were visualized based on side scatter area (SSC-A) versus forward scatter area (FSC-A). Then, single cells were gated based on forward scatter height (FSC-H) forward scatter area (FSC-A). From the single cell population, neutrophils were gated based on side scatter area (SSC-A) versus forward scatter area (FSC-A). Live neutrophils were gated based on lack of PI staining detected in Comp-PE_CF594-A. Finally, the mean fluorescent intensity (MFI) of live neutrophils was determined based on detection in Comp-FITC_BB515_A. (B, C). Anti-fibronectin and anti-β-actin antibodies were tested on Western blots of bovine MDEC CM undiluted, diluted 1:2, and diluted 1:4, for validation as reference proteins. Band densities relative to controls were graphed (i) and representative images of blots are shown (ii). Gray dotted lines on graphs indicate the control conditions expressed as 100%. Data points on graphs represents the results from one experiment, assessing the effects of CM from one MDEC line on 3 neutrophil preparations. n = 3 experiments. [file Image_1.tif]

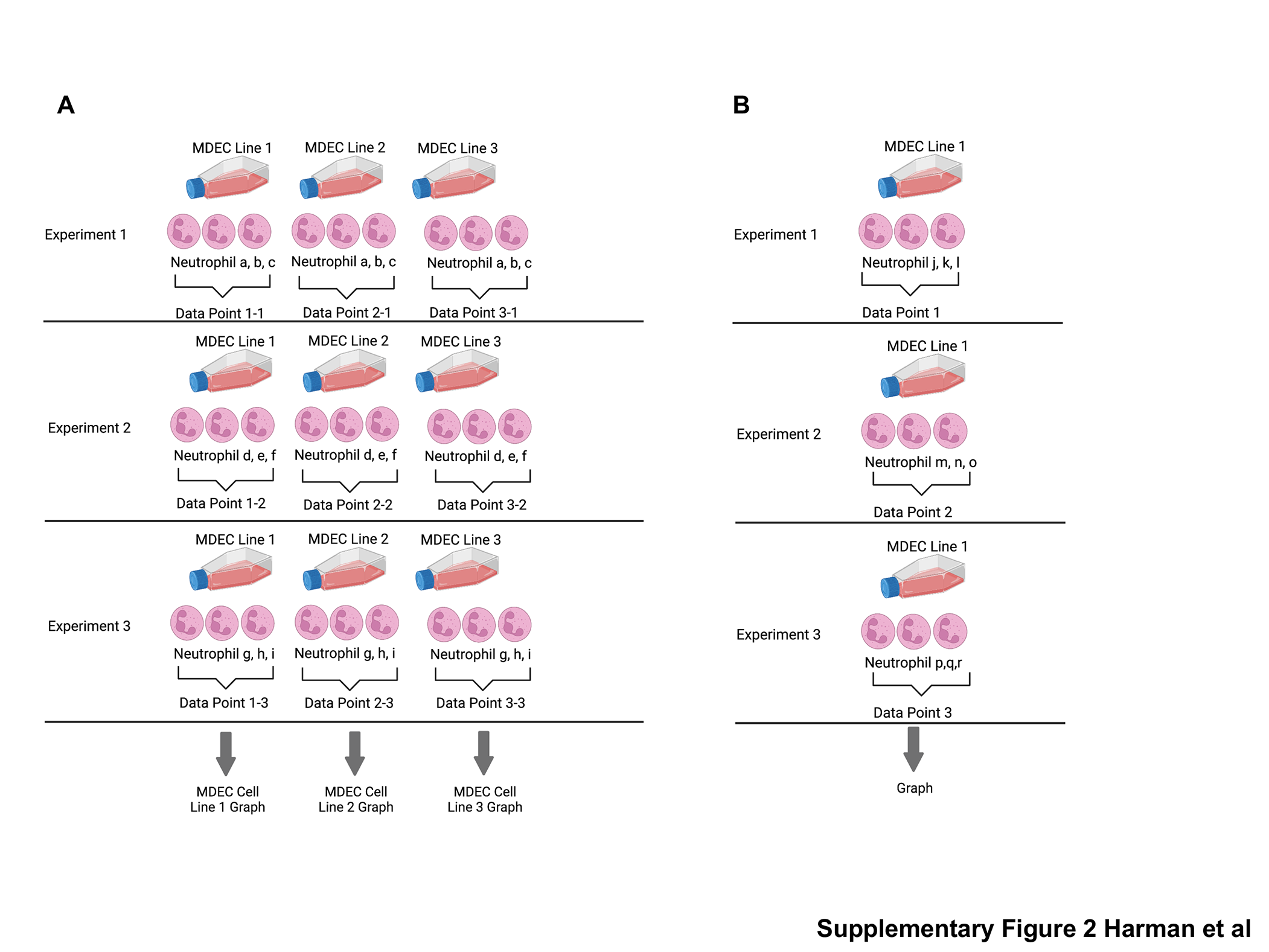

Supplement: Supplementary Figure 2 — Schematic of experiments and presentation of data. (A). The initial chemotaxis, phagocytosis, ROS production and NE activity assays were run as 3 experiments, each on a different day. For an experiment, MDEC CM was collected from 3 MDEC cell lines (1, 2 & 3) and tested against neutrophils isolated from the blood of 3 cows. Within an experiment, data from the 3 neutrophil isolations treated with CM from each MDEC cell line were averaged to create the data points shown on the cell line specific graphs presented in Figures 1 , 2 ; Supplementary Figures 3 - 5 . (B). Viability, active caspase detection assays, intra-versus extracellular DNA analysis, as well as RT-PCR, Western blots, chemotaxis, phagocytosis, and ROS production assays designed to determine which bioactive factors in MDEC CM were responsible for these functional effects on neutrophils were run as 3 experiments. Each experiment consisted of MDEC CM collected from one cell line, tested against neutrophils isolated from the blood of 3 cows. Data from the 3 neutrophil preparations used for each experiment were averaged to create the data points shown on the graphs presented in Figures 4 , 6 , 7 ; Supplementary Figures 1 , 2 , 6 . Image generated by Biorender. [file Image_2.tif]

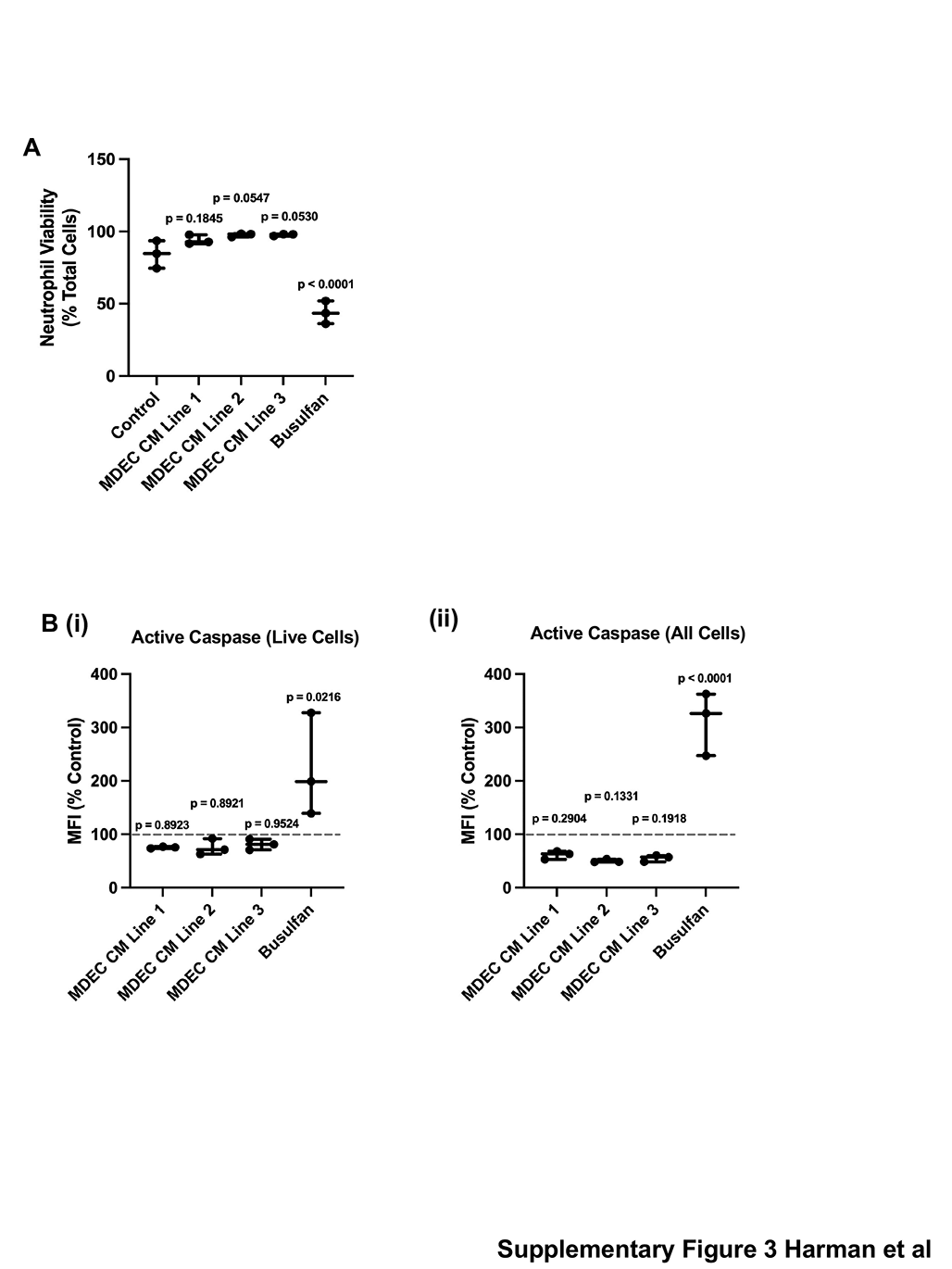

Supplement: Supplementary Figure 3 — Bovine mammosphere-derived epithelial cell (MDEC) conditioned medium (CM) does not induce neutrophil cell death or apoptosis. (A). Viability of neutrophils 6 hours (h) post isolation as detected by propidium iodide (PI) staining. Neutrophils were incubated for 1 h in control medium, MDEC CM from 3 cell lines or busulfan immediately after isolation, then maintained in control medium for 5 h before flow cytometric analysis. (B). Active caspase activity in neutrophils 6 h post isolation as detected by TF2-VAD-FMK Neutrophils were incubated for 1 h in control medium, MDEC CM from 3 cell lines or busulfan immediately after isolation, then incubated with TF2-VAD-FMK for 5 h before flow cytometric analysis. Analysis of live neutrophils (i). Analysis of all neutrophils (ii). Gray dotted lines on graphs indicate the control conditions expressed as 100%. Data points on graphs represents the results from one experiment, assessing the effects of CM from one MDEC line on 3 neutrophil preparations. n = 3 experiments. [file Image_3.tif]

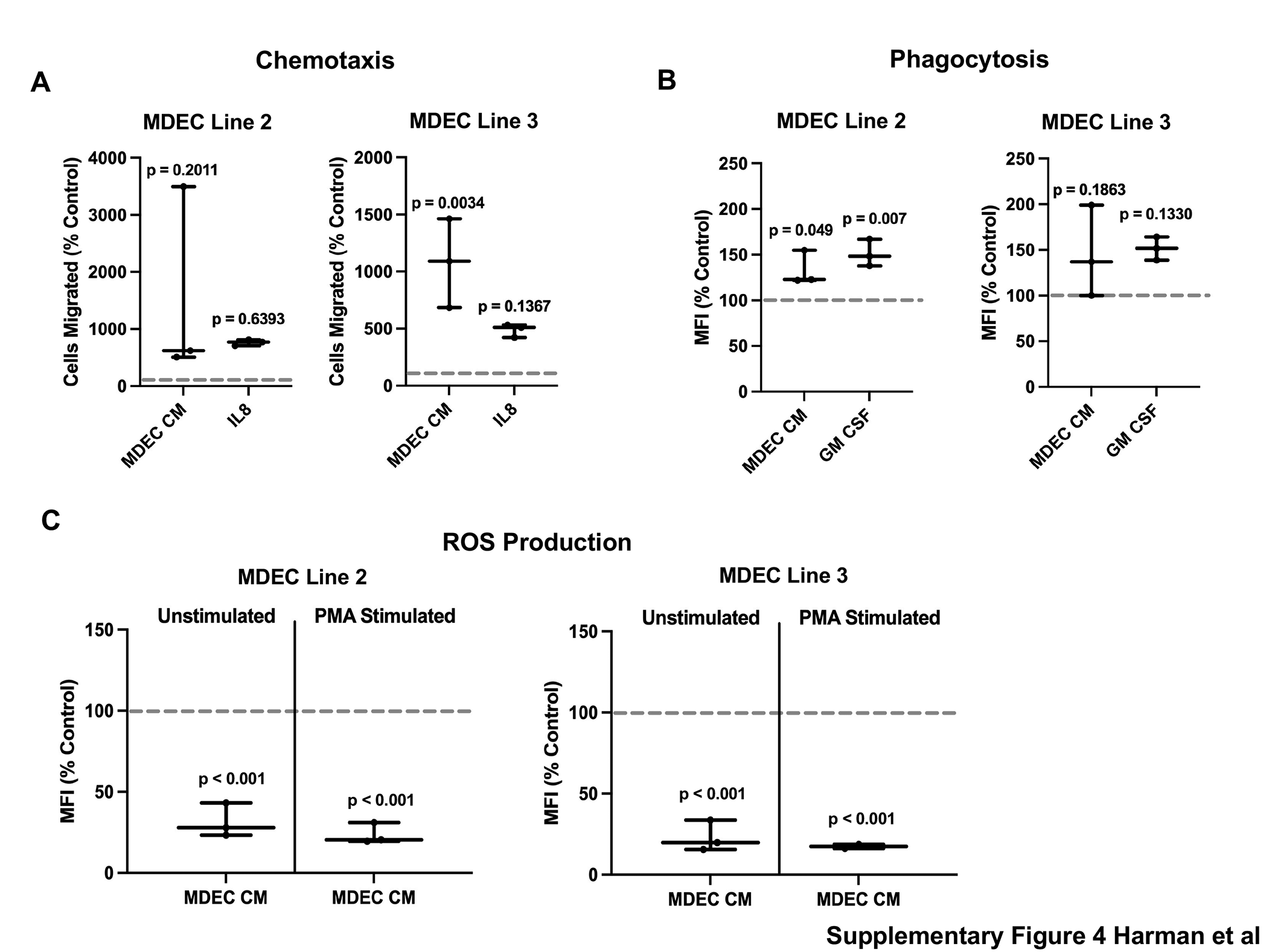

Supplement: Supplementary Figure 4 — Treatment of neutrophils with conditioned medium (CM) collected from multiple bovine mammosphere-derived epithelial cell (MDEC) lines leads to similar patterns of chemotaxis, phagocytosis, and reactive oxygen species (ROS) accumulation. (A). Bovine neutrophil chemotaxis was measured by counting cells that migrated through a mesh transwell insert into either RPMI control medium, CM from 2 different MDEC lines, or medium containing the chemoattractant interleukin-8 (IL8). Cells migrated, expressed as percent control are shown. (B). Neutrophil phagocytosis was determined by incubating cells with either RPMI control medium, CM from 2 different MDEC lines, or medium containing granulocyte-macrophage colony-stimulating factor (GM CSF). Labeled E. coli bioparticles were added and intracellular particles were detected by flow cytometry. Mean fluorescent intensities (MFI) expressed as percent control are shown. (C). Bovine neutrophil ROS production was quantified in neutrophils incubated in either RPMI control medium or CM from 2 different MDEC lines, plus or minus the stimulant phorbol myristate acetate (PMA). 2’,7’-Diochlorodihydrofluorescein diacetate (H2DCFDA) was added to the cultures and the intracellular oxidized form was measured by flow cytometry. Mean fluorescent intensities (MFI) expressed as percent control are shown. Each data point on graphs represents the results from one experiment, assessing the effects of CM from one MDEC line on 3 neutrophil preparations. Gray dotted lines on graphs indicate the control conditions expressed as 100%. n = 3 experiments. [file Image_4.tif]

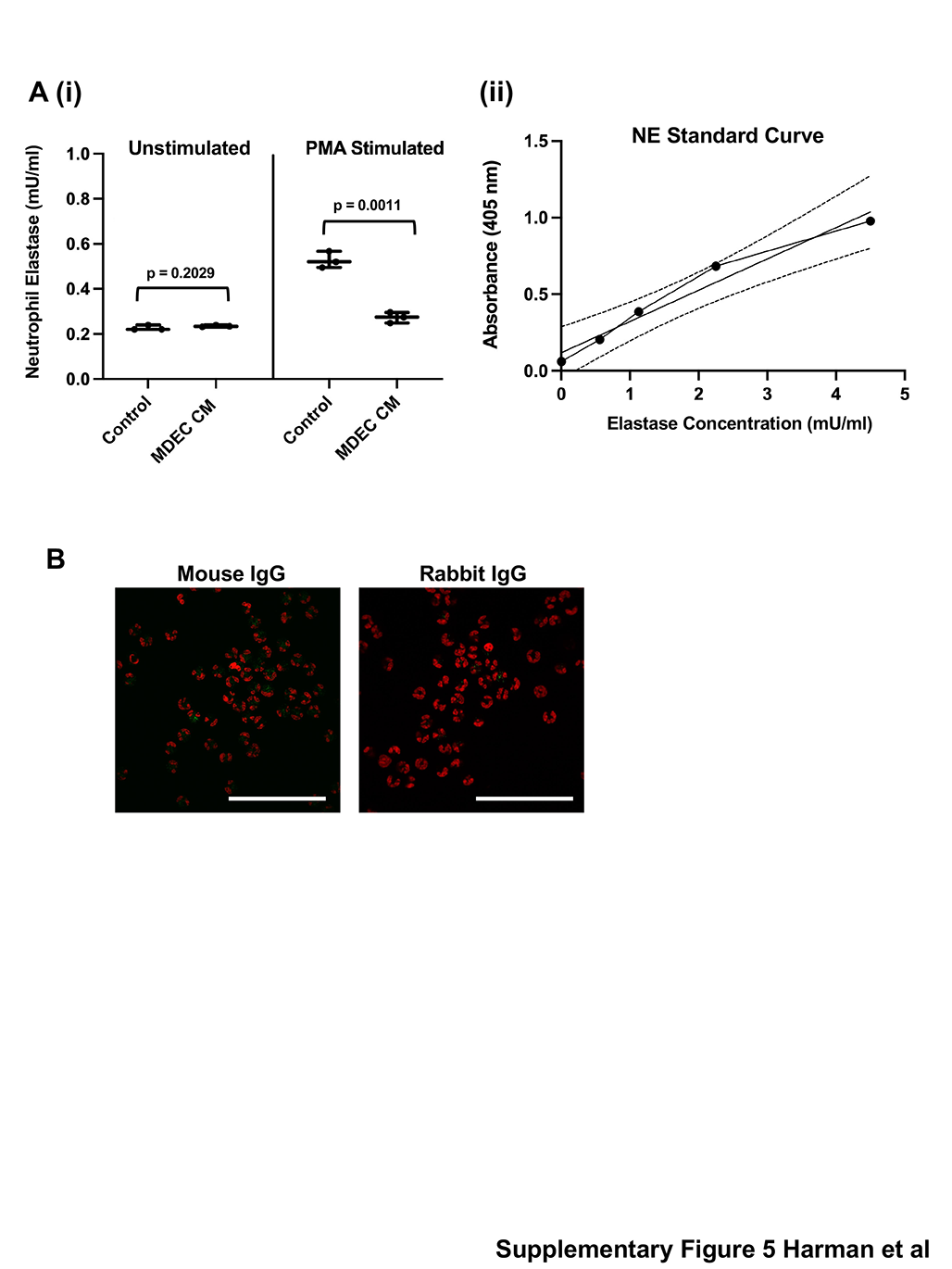

Supplement: Supplementary Figure 5 — Bovine mammosphere-derived epithelial cell (MDEC) conditioned medium (CM) suppresses neutrophil elastase (NE) secretion. (A). Neutrophils were incubated in either RPMI control medium or MDEC CM, plus or minus the stimulant phorbol myristate acetate (PMA), and secreted bovine NE was quantified via enzyme activity assays (i). Standards of known concentrations were included in the assay, and a curve was generated to quantify NE production in test conditions (ii). Each data point on graphs represents the results from one experiment, assessing the effects of CM from 3 MDEC lines on 3 neutrophil preparations. n = 3 experiments. (B). Images of neutrophils labeled with mouse or rabbit IgG as isotype controls for immunofluorescence assays. Mouse and rabbit IgG are labeled with a green fluorophore, SYTOX™ orange labels DNA. Scale bars = 100 µm. [file Image_5.tif]

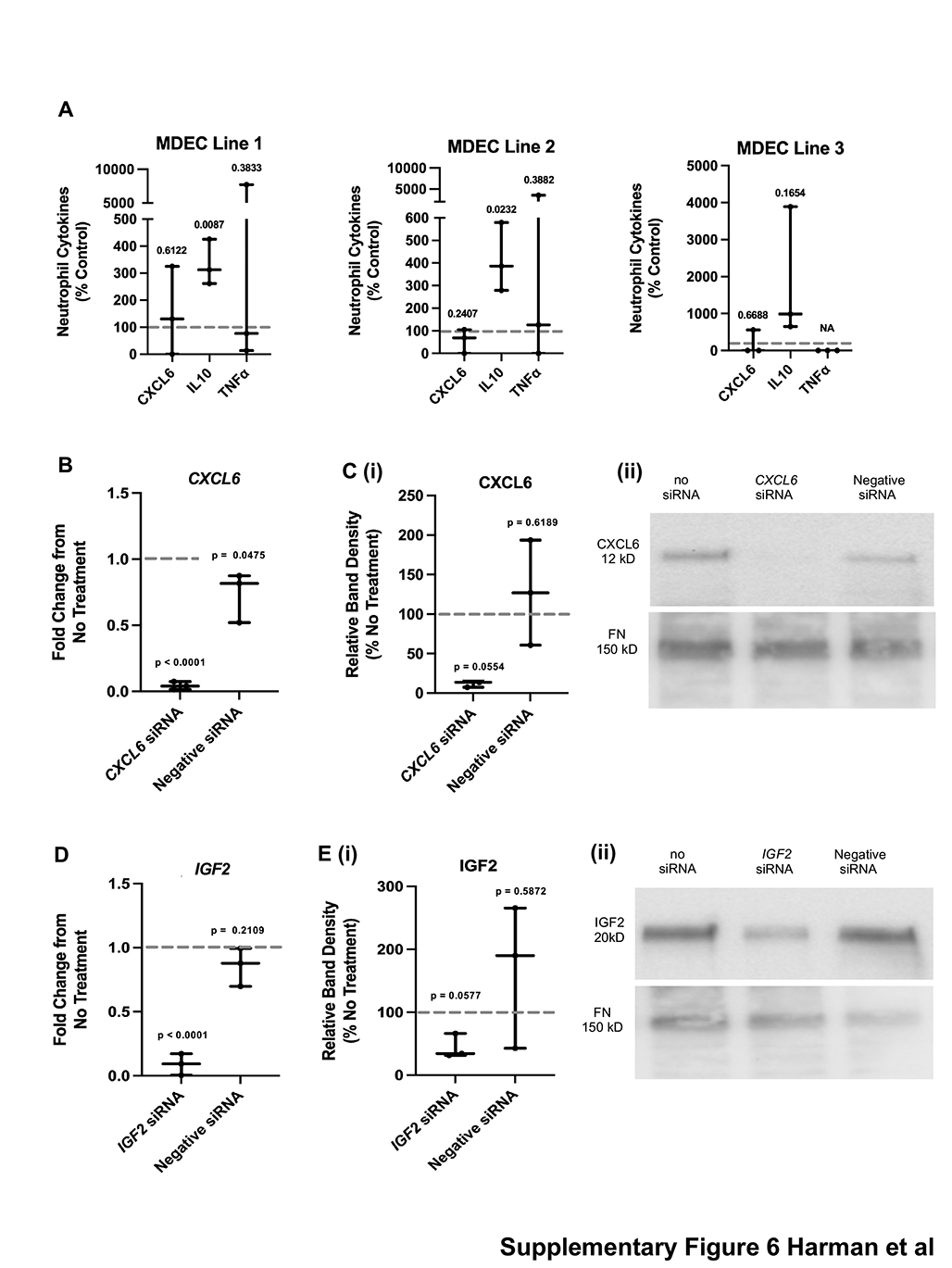

Supplement: Supplementary Figure 6 — Bovine mammosphere-derived epithelial cell (MDEC) conditioned medium (CM) variably influences neutrophil cytokine production and RNA-interference (RNAi) decreases chemokine (C-X-C motif) ligand 6 (CXCL6) or insulin-like growth factor 2 (IGF2) expression in bovine MDECs. (A). An enzyme-linked immunosorbent assay (ELISA) or fluorescent bead-based multiplex assay was used to measure the cytokines C-X-C motif chemokine 6 (CXCL6), interleukin-10 (IL10), tumor necrosis factor alpha (TNFα), and interferon gamma (IFNγ), in medium from neutrophils treated with CM from 3 MDEC Lines or control medium consisting of RPMI + 2% FBS. (B). Quantitative reverse transcription-polymerase chain reaction (qRT-PCR) was used to detect target transcript expression in MDECs transfected with either short interfering RNA (siRNA) against CXCL6 or a non-specific (negative) siRNA. (C). Western blots (WBs) were run to detect protein expression in MDEC CM collected from MDECs transfected with siRNA against CXCL6 or a negative siRNA. Band density as percent untransfected cells (no treatment) was calculated (i), representative images are shown (ii). (D). qRT-PCR was used to detect target transcript expression in bovine MDECs transfected with either siRNA against IGF2 or a negative siRNA. (E). WBs were run to detect protein expression in MDEC CM collected from MDECs transfected with either siRNA against IGF2 or a negative siRNA. Band density as percent untransfected cells (no treatment) was calculated (i), representative images are shown (ii). Gray dotted lines on graphs indicate the control conditions expressed as 100%. n = 3 replicates. [file Image_6.tif]

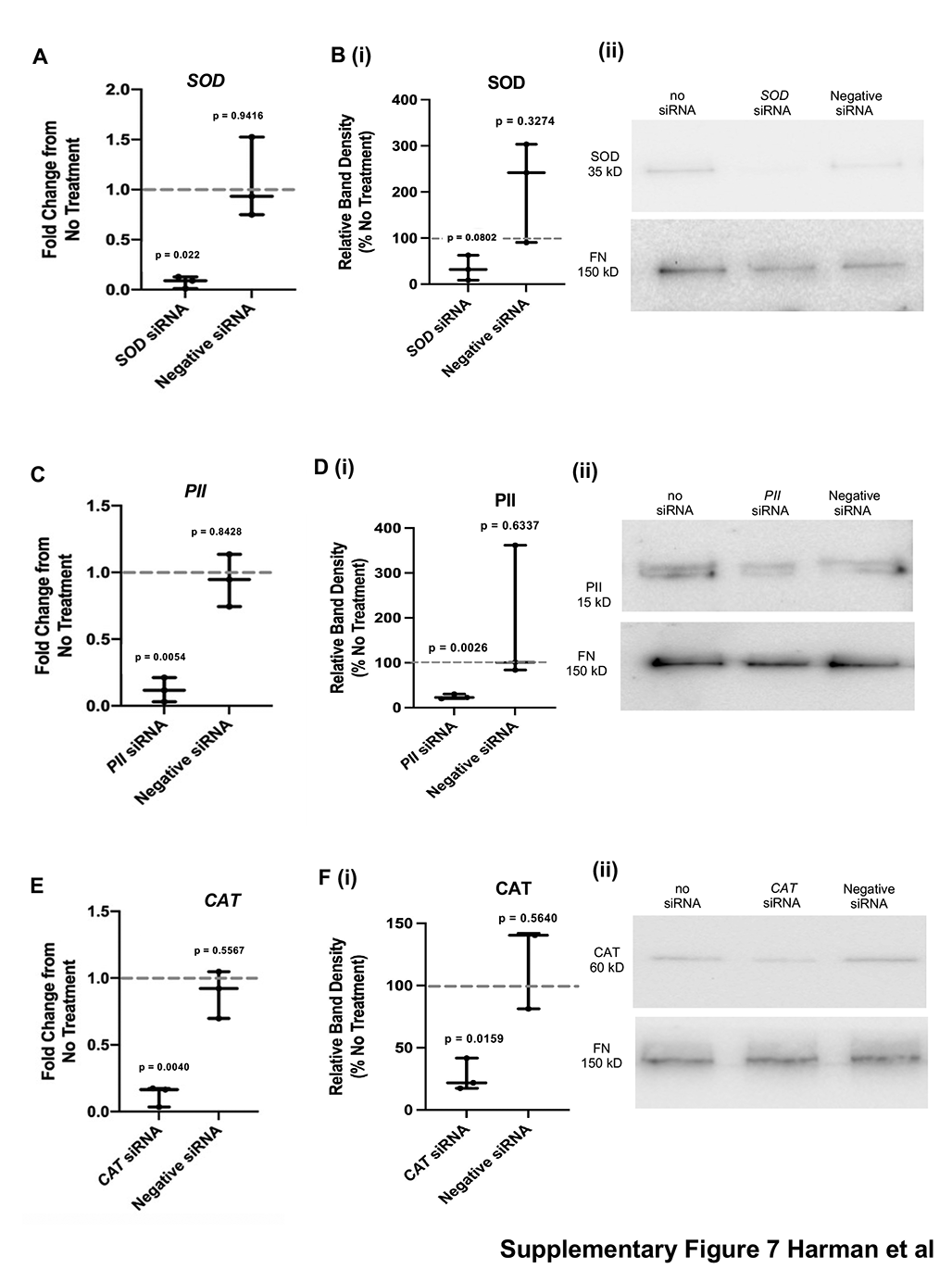

Supplement: Supplementary Figure 7 — RNA-interference (RNAi) decreases superoxide dismutase (SOD), peroxiredoxin 2 (PII), or catalase (CAT) expression in bovine mammosphere-derived epithelial cells (MDECs). (A). Quantitative reverse transcription-polymerase chain reaction (qRT-PCR) was used to detect target transcript expression in MDECs transfected with either short interfering RNA (siRNA) against SOD or a non-specific (negative) siRNA. (B). Western blots (WBs) were run to detect protein expression in MDEC CM collected from MDECs transfected with siRNA against SOD or a negative siRNA. Band density as percent untransfected cells (no treatment) was calculated (i), representative images are shown (ii). (C). qRT-PCR was used to detect target transcript expression in bovine MDECs transfected with either siRNA against PII or a negative siRNA. (D). WBs were run to detect protein expression in MDEC CM collected from MDECs transfected with either siRNA against PII or a negative siRNA. Band density as percent untransfected cells (no treatment) was calculated (i), representative images are shown (ii). (D). qRT-PCR was used to detect target transcript expression in bovine MDECs transfected with either siRNA against CAT or a negative siRNA. (D). WBs were run to detect protein expression in MDEC CM collected from MDECs transfected with either siRNA against CAT or a negative siRNA. Band density as percent untransfected cells (no treatment) was calculated (i), representative images are shown (ii). Gray dotted lines on graphs indicate the control conditions expressed as 100%. n = 3 replicates. [file Image_7.tif]

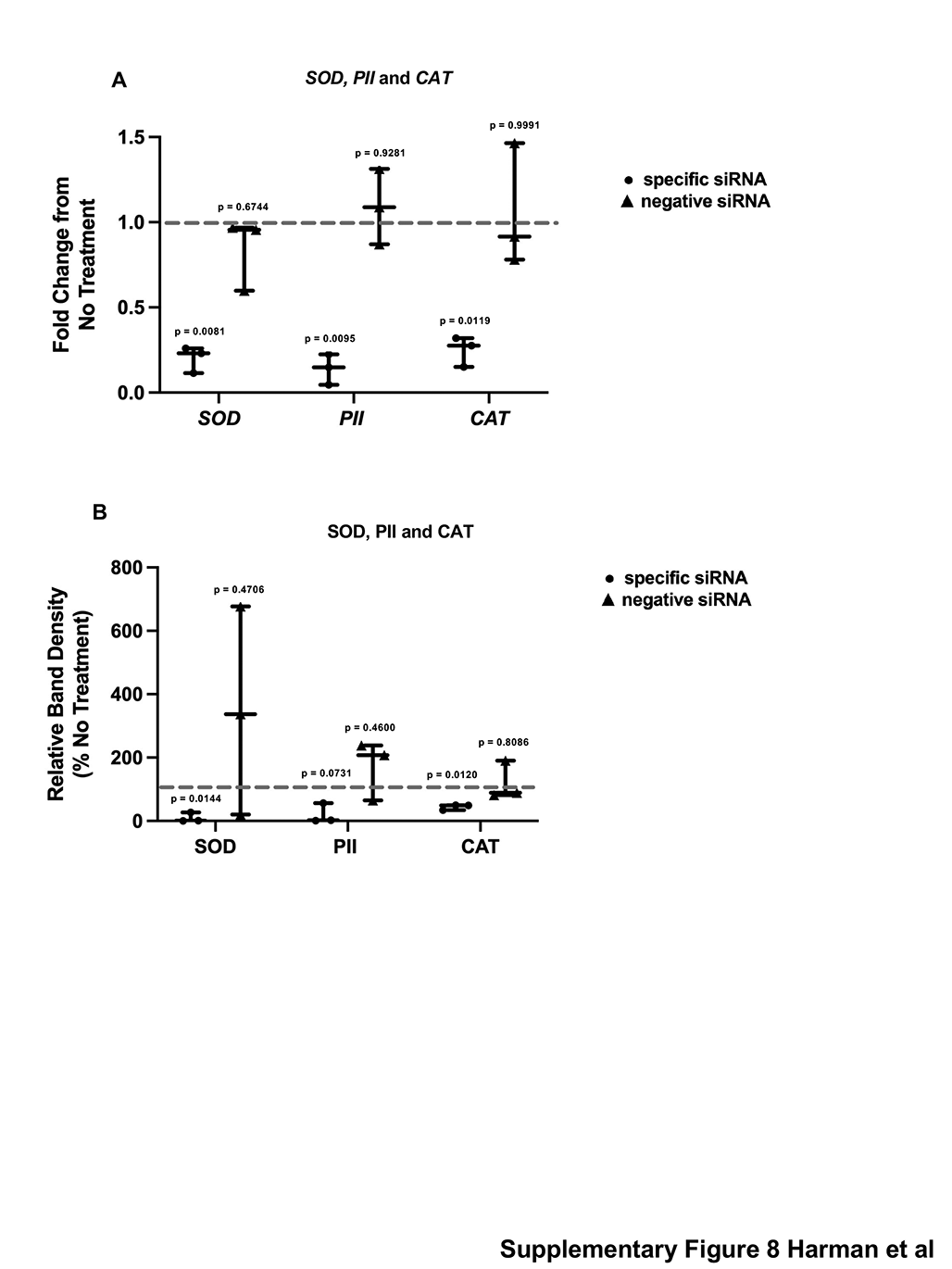

Supplement: Supplementary Figure 8 — RNA-interference (RNAi) decreases superoxide dismutase (SOD), peroxiredoxin 2 (PII), and catalase (CAT) expression in bovine mammosphere-derived epithelial cells (MDECs). (A). Quantitative reverse transcription-polymerase chain reaction (qRT-PCR) was used to detect target transcript expression in MDECs transfected with either short interfering RNA (siRNA) against SOD, PII and CAT or a non-specific (negative) siRNA at the same concentration. (B). Western blots (WBs) were run to detect protein expression in MDEC CM collected from MDECs transfected with siRNA against SOD, PII and CAT or a negative siRNA at the same concentration. Band density as percent untransfected cells (no treatment) was calculated. Gray dotted lines on graphs indicate the control conditions expressed as 100%. n = 3 replicates. [file Image_8.tif]
